# The role of geographic information system and global positioning system in dementia care and research: a scoping review

**DOI:** 10.1186/s12942-022-00308-1

**Published:** 2022-08-04

**Authors:** Neda Firouraghi, Behzad Kiani, Hossein Tabatabaei Jafari, Vincent Learnihan, Jose A. Salinas-Perez, Ahmad Raeesi, MaryAnne Furst, Luis Salvador-Carulla, Nasser Bagheri

**Affiliations:** 1 Department of Medical Informatics, School of Medicine, Mashhad University of Medical Sciences, Mashhad, Iran; 2 École de Santé Publique de L’Université de Montréal (ESPUM), Québec Montréal, Canada; 3 Visual and Decision Analytics Lab, Health Research Institute, Faculty of Health, University of Canberra, Canberra, Australia; 4 Health Research Institute, University of Canberra, Building 23 Office B32, University Drive, Bruce, Canberra, ACT 2617 Australia; 5 Department of Quantitative Methods,, Universidad Loyola Andalucía, Spain Faculty of Medicine, University of Canberra, Canberra, Australia; 6 Mental Health Policy Unit, Health Research Institute, Faculty of Health, University of Canberra, Canberra, Australia; 7 Menzies Centre for Health Policy and Economics, Faculty of Medicine and Health, University of Sydney, Sydney, Australia

**Keywords:** Dementia, Geographic information system, Spatial analysis, Global positioning systems, GPS, GIS

## Abstract

**Background:**

Geographic Information System (GIS) and Global Positioning System (GPS), vital tools for supporting public health research, provide a framework to collect, analyze and visualize the interaction between different levels of the health care system. The extent to which GIS and GPS applications have been used in dementia care and research is not yet investigated. This scoping review aims to elaborate on the role and types of GIS and GPS applications in dementia care and research.

**Methods:**

A scoping review was conducted based on Arksey and O’Malley’s framework. All published articles in peer-reviewed journals were searched in PubMed, Scopus, and Web of Science, subject to involving at least one GIS/GPS approach focused on dementia. Eligible studies were reviewed, grouped, and synthesized to identify GIS and GPS applications. The PRISMA standard was used to report the study.

**Results:**

Ninety-two studies met our inclusion criteria, and their data were extracted. Six types of GIS/GPS applications had been reported in dementia literature including mapping and surveillance (n = 59), data preparation (n = 26), dementia care provision (n = 18), basic research (n = 18), contextual and risk factor analysis (n = 4), and planning (n = 1). Thematic mapping and GPS were most frequently used techniques in the dementia field.

**Conclusions:**

Even though the applications of GIS/GPS methodologies in dementia care and research are growing, there is limited research on GIS/GPS utilization in dementia care, risk factor analysis, and dementia policy planning. GIS and GPS are space-based systems, so they have a strong capacity for developing innovative research based on spatial analysis in the area of dementia. The existing research has been summarized in this review which could help researchers to know the GIS/GPS capabilities in dementia research.

**Supplementary Information:**

The online version contains supplementary material available at 10.1186/s12942-022-00308-1.

## Background

Over 55 million people are estimated to live with dementia worldwide, with an increase of nearly 10 million new cases yearly [1]. This places a significant financial burden on health care systems and societies. The cost of caring for people with dementia was estimated about 818 billion US dollars (about 1.1% of global gross domestic product) worldwide in 2015 and is estimated to be more than two trillion US dollars by 2030 [1]. Given the growing socioeconomic impacts of dementia, many studies have focused on factors, from individual to environmental, associated with the development of this disorder [2–4]. Although understanding these associations is essential for the development of treatment and preventive strategies [3], understanding the spatial pattern of dementia over space and time using the Geographic Information System (GIS) and Global Positioning System (GPS) is essential for designing tailored interventions at the population level [5].

GIS is a kind of information systems that can be used to manage, analyze, and visualize geographic information. The first applications of GIS in health, more defined as spatial analysis, was creating a map to visually represent cholera outbreaks across 48 Paris districts in 1832 [6] and John Snow's work to map and  visualize the source of a cholera outbreak in London in 1854 [7]. Roger Tomlinson developed the first computerized GIS in 1963 [8]. These computerized systems have continued to be applied in public health research to understand the distribution of diseases. GIS has a unique capacity to link data from different sources (e.g., individual, locational and organizational databases) and identify and visualize geographic variations in disease patterns over time and space [5, 9]. In addition, policymakers can use GIS capabilities to quantify the interaction between different levels of dementia care and design geographically targeted interventions [5, 10].

Recently, GIS has been used for dementia research by combining spatial and non-spatial data into one framework to analyze the dementia care system [10, 11]. For instance, researchers used GIS to study high-risk areas by identifying potenteil spatial autocorrelation in the distribution of dementia patients [12]. Furthermore, some studies used explanatory spatial analysis to identify associations between dementia occurrence and environmental risk factors [13]. GIS can also be used to measure spatial accessibility to healthcare services [14], determining the spatial distribution of medicines [15] or services needed by patients [16].

GPS is a space-based navigation system that collects and provides data about positioning, navigation, and timing [17]. GPS was originally intended for use in the military but then widely used in healthcare, especially for mental health disorders, to monitor, follow, track and manage the care process of patients [18, 19]. GPS assesses and analyses patients’ out-of-home and driving behavior [20–25]. Out-of-home behavior patterns can predict cognitive impairment disorders [21]. Some studies use GPS to collect activity data to detect real-time disoriented behavior and the uniformity of a patient’s walking. GPS has also been used as real-time navigation assistance [26]. Collected data by this system defines the activity space of patients to assess their social health and independent activity [27].

Many studies used GIS and GPS in health research [28]. The different GIS/GPS applications in dementia field emphasize the need to systematically synthesize and summarize the relevant studies. This can contribute to identify the potential gaps in the utilization of these space-based systems in dementia care and research. However, there is no review article on using these systems in dementia context. Therefore, this scoping review aims to synthesize the literature, understand the role of GIS and GPS in dementia care and research and elaborate on their applications in this field. Our results can help researchers to know the different capabilities of GIS and GPS in dementia setting.

## Methods

We implemented the scoping review method according to Arksey and O’Malley’s methodological framework and the PRISMA standard [29]. Arksey and O’Malley’s methodological framework provides a guideline for conducting a scoping review. It consists of five steps: identification of the research question, identification of the relevant studies, selection of included studies, charting the key elements, and summarizing and reporting the results [30]. The PRISMA is an evidence-based standard with minimum essential items used for reporting in systematic reviews and meta-analyses [31]. This study used PRISMA-ScR, a PRISMA extension intended to apply for scoping reviews (Additional file 1. PRISMA-ScR-Checklist). This standard, published in 2018, contains 20 essential and two optional items and helps to improve the reporting of scoping reviews [32].

### *Identifying the research question*

What are the different applications of GIS and GPS in dementia care and research, and what methodologies based on GIS and GPS were used in dementia literature?

### *Identifying the relevant studies*

#### *Search strategy*

A comprehensive search strategy was developed by combining the related keywords to retrieve all dementia and Alzheimer’s studies using a GIS/GPS approach. Additionaly,  a set of MeSH terms were applied in the search strategy to implement a broad search in PubMed. The  concepts taken in this review are summarized in Table 1, and the complete search strategy for each database is provided in Additional file2. Search strategy.

**Table 1:** Comprehensive set of concepts used in search strategy

Subject	Concepts
GIS/GPS approaches	Geographic Information Systems (GIS), Global Positioning Systems (GPS), satellite Imagery, remote Sensing, mapping (choropleth map, heat map, dasymetric map), spatial Analysis (Spatio-Temporal analysis, spatial autocorrelation, spatial regression, Hotspot analysis, spatial clustering, geographic cartography, georeferencing, spatial accessibility, global system for mobile communications (GSM)
Dementia	Dementia, Alzheimer’s disease, neurocognitive disorder, cognitive impairment, cognitive decline, cognitive dysfunction, mental deterioration, tauopathies, wandering, getting lost, patient tracking.

#### *Information sources*

PubMed, Web of Science, and Scopus were searched to retrieve articles published up to June 06, 2022, with no geographic restrictions and time limitations. Searches were limited to English studies and focused on human subjects at individual and population levels. The results were imported into Endnote X8 reference management tool (Clarivate Analytics, Philadelphia, PA, USA), and duplicate references were removed from the results.

**Fig. 1:**
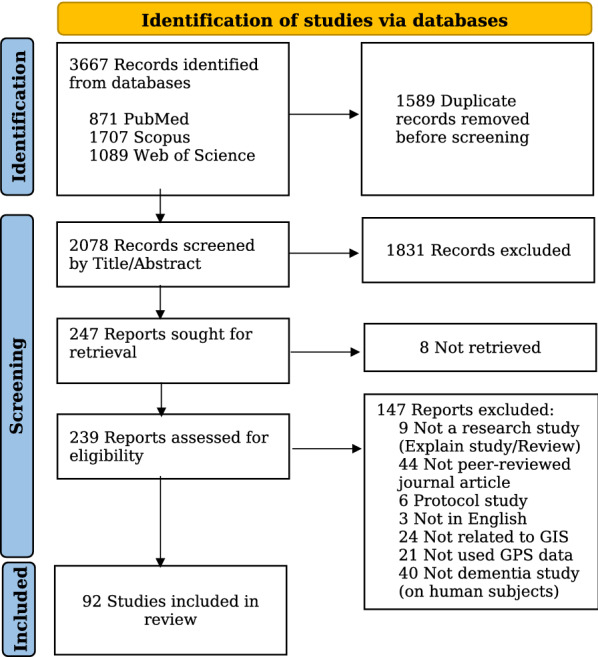
Flowchart of study selection. Excluded reports may have had more than one reason for exclusion, but only one reason was listed here

The search strategies identified 3667 citations from the three databases. After removing duplicates and excluding irrelevant articles based on title and abstract screening, 239 articles remained for an in-depth review, of which 147 did not meet the inclusion/exclusion criteria. Finally, 92 studies were included and considered for data extraction (Fig. 1) (Additional file 3. Extraction Table).

### *Selection studies*

#### *Eligibility criteria*

Original studies published in peer-reviewed journals which  written in English were considered in this study.

Studies were eligible for inclusion if they met one of the following criteria:

1. Applied at least one GIS approach in dementia or severe cognitive impairment (if it had been clinically diagnosed as dementia).
2. Studies focused on GPS technology if dementia/Alzheimer patients had used the GPS, and the gathered data by GPS was analyzed.

Studies were excluded if:

1. They only assessed the attitudes of patients, their families, or professional caregivers toward using GPS-based technology.
2. They only assessed the ethical aspects of using GIS/GPS.
3. They focused on system design and development, technology acceptance, user experience, usability, and utility.
4. The interview or questionnaire-based studies used neither a GIS approach nor any analysis regarding GPS data.
5. Studies only used sensors to track people with dementia and did not use any GIS approaches.
6. The studies without any human participants or considering only healthy volunteers without any patients as their subjects or focused on birds, rats, and other animals.

The reviews and non-peer-reviewed literatures such as editorials, conference or congress papers, book sections, and study protocols were excluded from the study.

#### *Screening process*

The expert panels of the authors with expertise in GIS, spatial analysis and Alzheimer’s disease were held. The meetings were set up for study design, conceptualization, category definition, methodology, and data extraction. At the first screening step, the authors independently evaluated the title and abstract of retrieved articles (NF, HT, MF, JS, VL). Irrelevant studies were excluded against the mentioned criteria. Then screened articles were double-checked by the first author, and if she had any doubts, a meeting was organized and the consensus opinion was considered and resolved by group discussion. Finally, the full text of potentially relevant articles was reviewed, and eligible studies were identified for data extraction.

### *Charting the data*

A checklist was designed for data extraction (see data items part). Six authors (NF, HT, BK, AR, JS, VL) extracted the data independently. The first author aggregated the extraction results, and a double-check was performed by her.

To extract GIS applications, the first author prepared an initial list based on two similar studies [7, 33]. This list was assessed and discussed  in the meetings and includes four main categories; disease mapping, planning, access and care, and risk factor analysis. In this list, the “other applications” category was considered to record different items that were impossible to assign to the four groups. After data extraction, expert panel members assessed and discussed recorded items in this category in meetings. If these items could be placed in the main categories, they had been assigned to them, such as disease surveillance assigned to the disease mapping category. However, the rest of the items were labelled as two new categories; “basic research” and “data preparation”. Finally, the authors discussed and agreed on the final list. Thus, six categories of GIS/GPS applications were considered in this study.

#### Data items

For each eligible article, the following data items were extracted: title, author (s), year of publication, study location (country), study populations, outcome measures, intervention type, geographical level of analysis (scale), the aim of the study, main results (GIS-related key findings), type of GIS/GPS methodology, geospatial analysis technique/ technology, type of GIS/GPS application. Each study could use more than one method and technique and had more than one application. The categories of GIS/GPS methodology were classified into five types.

Types of GIS methodology:

- ***Thematic mapping***: A type of map that visualizes invisible spatial patterns of a phenomenon or provides some information about a geographical area. This study includes several kinds of maps, such as inset map, choropleth map, heat map, and buffer map [34].
- ***Spatial modelling***: Constructing models to comprehensively analyze spatial and non-spatial properties [34, 35]. Spatial modelling includes spatial agent-based modelling [36–38], spatial regression modelling [39], and spatial network analysis [40, 41].
- ***Web GIS***: An architectural approach that provides a platform for spatial data analysis and spatial pattern visualization by building interactive web maps such as online spatial dashboards, interactive mapping, or online mapping [42, 43].
- ***GIS/GPS tools and technology***: This category includes a wide range of tools and technologies for gathering, analyzing and combining spatial data, such as GPS, a navigation system providing location and time information [44].
- ***Space-time clustering***: A spatial analysis to identify clustering of spatial patterns in a geographic area over time [45–47], such as Space-Time Scan Statistics [48].

### *Collating, summarizing, and reporting results*

The geographical distribution of the included studies was visualized in ArcGIS Desktop software (ESRI, Redlands, CA, USA). Microsoft Excel was employed to create appropriate charts and tables to summarize the results.

## Results

**Fig. 2:**
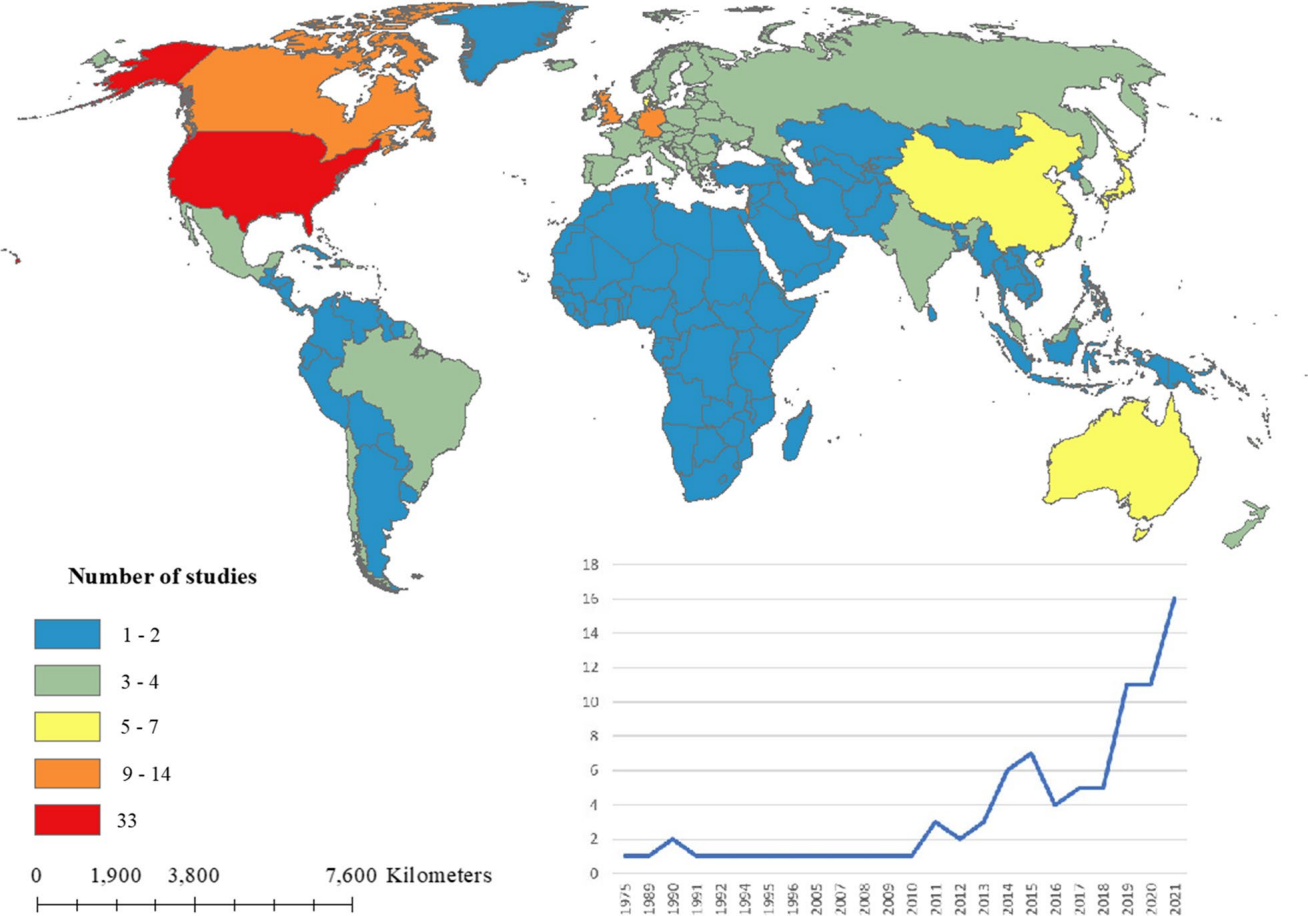
Geographical variation of included studies and number of publications over time

Of 92 eligible studies, 11 articles used data from two or more countries [12, 21, 49–57]. Two studies applied worldwide data [55, 56] and one used only continental Europe data [57]. Most studies reported data from the United States (n = 33), the United Kingdom (n = 14) and Germany (n = 11). The first study was published in 1975, and between 1975 and 2010, only 14 studies were published. However, the number of studies rapidly increased, and 78 studies were published during 2011–2022 (Fig. 2).

### GIS/GPS methodologies in dementia care and research

**Fig. 3:**
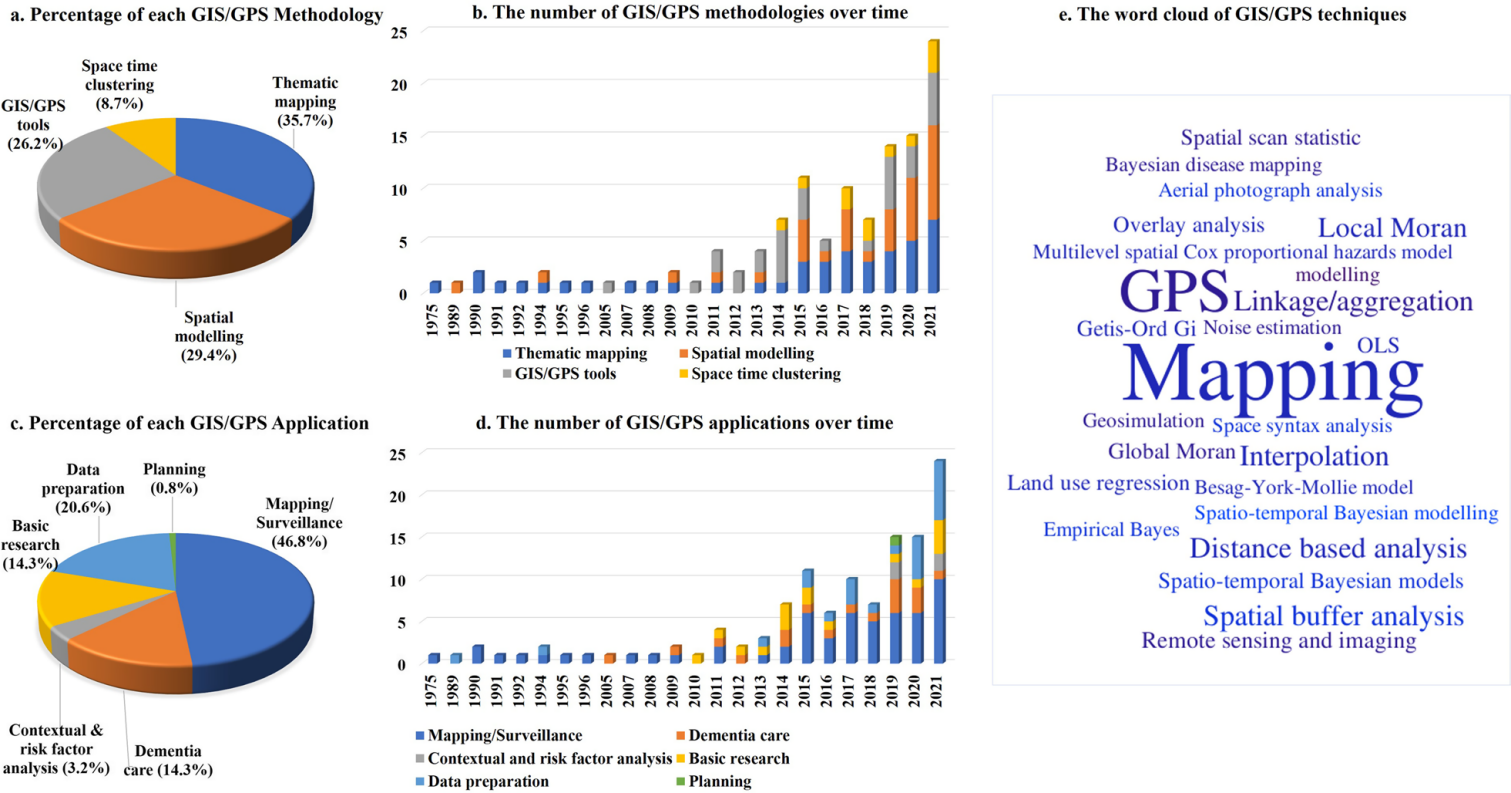
GIS/GPS methodologies and applications

The methodologies applied in the included studies were categorized as thematic mapping (n = 45), followed by spatial modelling and analysis (n = 37), GIS/GPS tools and technologies (n = 33), and space-time clustering (n = 11) (Fig. 3a). No study employed web GIS methodology for data analysis and visualization.

As shown in Fig. 3b, the usage of GIS methodology in dementia has increased over time, particularly since 2014. Thematic mapping was the earliest methodology used in dementia; there were only 14 studies between 1975 and 2014, but its use has increased rapidly since 2015. The focus on spatial modelling and analysis has increased since 2015. The initial use of space-time clustering goes back to 2014, and the usage trend did not change over time.

Different types of techniques were used in each category (Additional file 4. Techniques). All these techniques are listed and weighted based on frequency in Fig. 3e. As Figure 3 shows, mapping and GPS, which belong to the thematic mapping and GIS/GPS technology categories, respectively, were used frequently and much more than other techniques in dementia research. Note that we have not shown the number of studies for 2022 in figure 3 as our search strategy was conducted in the middle of 2022 and due to incomplete data of 2022, the time trend will not be correct.

### GIS/GPS applications in dementia care and research

Six main domains of GIS/GPS applications in dementia research were identified including mapping and surveillance (n = 59), data preparation (n = 26) dementia care (n = 18), basic research (n = 18), contextual and risk factor analysis (n = 4), and planning (n = 1) (Fig. 3c). GIS mainly was used for thematic mapping from 1975 to 2010, but the GIS approaches and the frequency of GIS applications have increased since 2011. GIS/GPS techniques have been used in basic research since 2011 and for policy planning purposes in 2019 (Fig. 3d). A comprehensive definition for each group of applications is provided in Table 2.

**Table 2:** Comprehensive definition of each GIS/GPS application in dementia care and research

GIS/GPS application	Definition
Disease mapping or surveillance	Studies containing any spatial mapping to depict the geographical distribution of dementia outcomes or any other related phenomena (58) ranging from simple descriptive maps to more analysis-based clustering maps. Also, disease surveillance refers to more comprehensive information about dementia and conditions which can be used to evaluate and interpret the effectiveness of preventive interventions and control health measures in the public health contexts (59).
Dementia care	Using GIS/GPS tools or technologies or other spatial approaches such as descriptive mapping in dementia care or rehabilitation to support care, such as GPS monitoring system to locate missing patients (60) or managing patients’ health activity (61). Also, this category includes articles that focus on measuring spatial accessibility to dementia care services. The spatial accessibility describes a population’s ability to reach to health centers and facilities when needed (62). This category focus on the direct role of GIS approaches in the care of patients.
Contextual/risk factor analysis	Examining the spatial relationship of any environmental or contextual factors with dementia (63).
Planning	The studies identify patients’ needs and issues with resource allocations and develop health plans specific to the study area (64).
Basic research	Not-applied studies aim to assess patients’ behavioral patterns (26), out-of-home mobility (65), or other concepts to understand the patients situation.
Data preparation	GIS/GPS approaches are used to prepare data for non-spatial modellings. For example, interpolation (66) and buffer analysis (67) have measured exposures data for non-spatial modellings.

#### Mapping and surveillance

The studies in this category focused on any visualization for dementia-related outcomes. Most maps were applied to visualize the spatial and temporal patterns of dementia/Alzheimer’s mortality [49, 50, 55, 68–78] and hospital admission rates [79]. Some visualized spatial distribution of birthplace of dementia patients [80, 81], the area with high concentration [82], incidents of missing patients with dementia [83], dementia risk/rate [4, 12, 57, 84–93], hospice use [94], opioid use [95], and antipsychotic drug use [15]. Some studies used mapping in preparing exposure data [67], arsenic concentrations [96], ozone levels [97], spatial distributions of air pollutants [98] and ranking countries in medical tourism [56]. Three studies mapped access to services [14, 16, 99], and only one used resource allocation related to the care of dementia patients [64]. Also, one study used mapping to visualize patients’ mobility patterns [100].

#### Data preparation

The studies in this category mainly used spatial analyses to prepare data for non-spatial models. These spatial analyses include spatial interpolation [57, 66, 81, 96–98, 101, 102], buffer analysis [54, 67, 102–105], overlay analysis [97], land-use regression [4, 106], satellite imaging [107], aerial photograph analysis [108], space syntax analysis [109], noise estimation [110], spatial linkage and aggregation [75, 87, 89, 111, 112], distance-based analysis [113]. These techniques were used to measure environmental factors such as air pollutant exposures [4, 57, 66, 97, 98, 101, 102, 106, 107], green and blue spaces [54, 102, 104, 105, 112], organophosphorus (OP) exposure [67], aluminum concentration [81], hilliness [103] and arsenic level [96]. Furthermore, the studies calculate sidewalk coverage [108] and neighborhood integration [109], estimate noise origination [110] and construct an area deprivation index [111]. Other applications were quantifying and preparing sociodemographic variables and risk factors at the statistical area level [87, 89, 93] and distance-based adjusting rates [113].

#### Basic research

This category includes basic research (not-applied studies) that assess and compare behavioral patterns [26], out-of-home mobility [65], or other concepts to understand the patients’ situation better. Eighteen studies were identified in this category, and all applied GPS as a space-based technology to gather tracking data in exploring patients’ behavior patterns. In most studies, analysis and interpretation of spatial GPS data were used to assess patients’ out-of-home behavior [20–23], mobility patterns [52, 53, 65, 114–117], life-space metrics [118, 119], and driving behavior [24, 25]. In one study, GPS data were used to propose a Bayesian classifier model to estimate the probability of wandering [120].

Another study in this category assessed GPS-based data quality and validity based on compliance rates of the participants with study protocol and requirements [121]. Further, a study compared behavioral competence and the emotional well-being of mild cognitive impairment adults with a healthy group and persons with dementia. This study identified more similarities in mild cognitive impairment adults’ behavior compared to the dementia group [51].

#### Contextual and risk factor analysis

Four studies used GIS models to investigate the association of contextual and environmental risk factors with dementia-related outcomes. A study used the Ordinary Least Squares regression model to test the potential association of neighborhood-level disadvantage with cognitive decline. Highly disadvantaged neighborhoods measured by Area Deprivation Index were associated with neurodegeneration and cognitive decline [122]. Another study applied spatial buffer analysis and ordinary least sqaure to explore the association between dementia-related missing incidents and outdoor landmarks. The high density of outdoor landmarks was identified as an environmental risk factor for getting lost [83]. The spatial Cox proportional hazards model assessed the relationship between exposure to air pollutants and PM2.5 with dementia incidence [106]. Further, satellite imaging analysis identified the correlation between the number of mining sites and dementia mortality in another study [123].

#### Dementia care

Eighteen papers applied GIS/GPS approaches in dementia care. Most studies in this category focused on GPS technologies (N = 12). They used GPS embedded in a smartwatch [61, 124], Personal Digital Assistant [125], mobile phone [126, 127], and other portable tracking devices [27, 124, 128–130]. Most applied GPS to get real-time health data to track and predict typical locations and movements and detect spatial disorientation patterns to provide appropriate assistive services [26, 124, 125, 127, 129, 130]. Some studies used data to explore activity space to examine social health [27], understand everyday life through socio-spatial relational care [100], understand the effect of caregivers burden on behavioral and emotional status of care recipients [128], and monitor and manage patients’ health and locations [61, 126]. In one of the studies, GPS was used to help people with mild Alzheimer disease to improve their driving performance and safety [131].

Five articles focused on health access using GIS approaches such as distance-based analysis [14, 16, 64, 99, 132] and geo-simulation [99] to measure spatial accessibility in the USA [16, 99], Ireland [14], Taiwan [64] and Japan [132]. Three studies examined accessibility to services [14, 16, 64] for patients and caregivers, and the other studies focused on accessibility to brain bank donation centers [99], and screening sites [132]. Only one study used descriptive mapping to visualize the geographical variation of drugs used in dementia care [15].

#### Planning

Only one study, conducted in Taiwan, fell in this category. Two new indicators were developed to facilitate dementia service allocations in this study. The indicators focused on supply and demand [64].

### Association of GIS/GPS applications with the corresponding methodologies

**Fig. 4:**
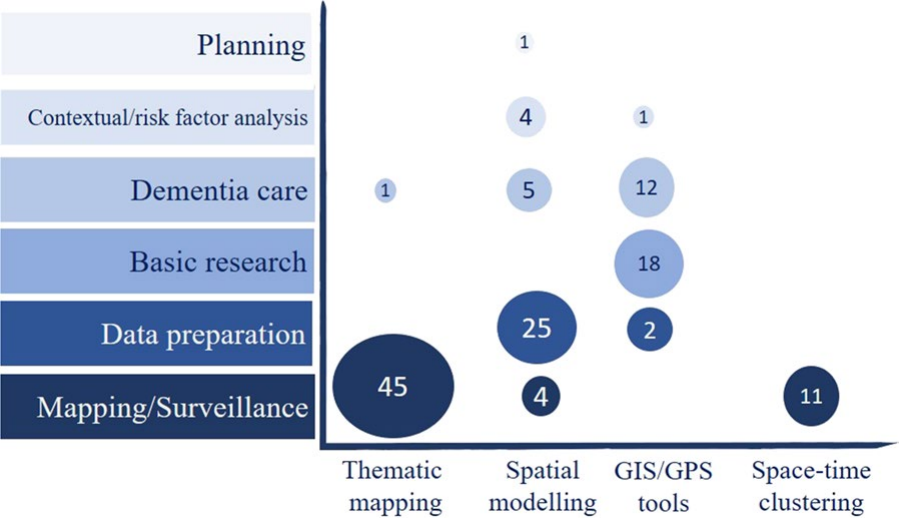
GIS/GPS applications associated with their corresponding methodologies

The main application of thematic mapping was for dementia epidemiology and outcomes mapping (n = 45). Only one study used thematic mapping in dementia care [15] to visualize geographical variations of antipsychotic drugs used by patients. Most studies applied spatial modelling in data preparation (n = 25) to measure and compute exposures for non-spatial modelling. A few studies used spatial modelling such as ordinary least squares in contextual factor analysis (n = 4), and only one study was directly conducted in dementia planning. GIS/GPS tools and technologies, the most common of which was GPS (Fig. 3e), were most commonly applied in basic research (n = 18) and dementia care (n = 112). Finally, space-time clustering was only used to map dementia-related conditions (Fig. 4).

## Discussion

This study was undertaken as a state-of-the-art study regarding dementia research and care. The study aimed to understand the role of GIS, geospatial analysis and GPS technologies in dementia care and research. To our knowledge, this scoping review is the first literature review in this field. We found 92 studies published from 1975 to 2022, demonstrating that the applications of GIS/GPS in dementia research have considerably grown over the past decade as GIS and GPS technology expanded and evolved. This study revealed six main applications of these systems in dementia care and research, including mapping or surveillance, dementia care and accessibility, contextual/risk factor analysis, planning, basic research, and data preparation. While GIS has been widely used to map dementia-related conditions, they have been used very little in service planning for dementia care.

Thematic mapping and GPS data collection are widely used in dementia research. Mapping was the most popular application to visualize geographical patterns of dementia-related conditions or outcomes in the study areas. The application of GPS as an advanced data collection tool has been more frequent in recent years compared to previous decades. It can be due to progress in telecommunication and hardware technologies such as smartphones which caused better accessibility to GPS in recent years. Dementia affects a patient’s functionality in daily life. They have severe problems in everyday activities, and more than 73% experience getting lost [133], so healthcare systems have focused on GPS-used devices for monitoring and tracking patients [61].

Our scoping review showed that GIS had been used very little in dementia care (14.3%) and planning (0.8%). The later finding is even less than in similar areas such as multiple sclerosis (MS) (4%) [33] and mental health care (15%) [134]. Policy planning and resource allocation are critical for optimal care of dementia across communities [135]. This is a real gap in the area of dementia. GIS has a unique capacity for identifying and visualizing unmet needs areas for policy interventions. GIS can reveal and visualize complex relationships between cases in both time and space. This visualization helps policymakers identify high-risk areas to implement the best response strategies. Location-based information supports healthcare needs assessment to resource management. In this capacity, public health officials can analyze and manage population-based health problems efficiently [136, 137]. GIS can support public health managers in making proper decisions and planning by addressing issues such as available health resource allocations [138], control and prevention of disease, and cost management [136]. Furthermore, GIS can link spatial and non-spatial characteristics of dementia care services/providers and visualize care provision across local areas and within different population groups. This will provide new knowledge for evidence-informed policy planning in dementia care and research.

Policymakers could use advanced space-time modelling/clustering to identify variations in service provision over space and time and design geographically targeted interventions. Furthermore, this research demonstrated that the use of GPS has increased in recent years, particularly in data collection for primary and secondary dementia care and even in rehabilitation services for dementia care and research. This capability can help researchers collect precise geo-linked data and in high-quality data preparation in dementia research. The utilization of GPS, for example, in monitoring the pattern of walkability in people living with dementia is an important application of space-based systems in the tertiary care of dementia across communities [26, 53, 116]. Additionally, researchers have been using advanced tracking systems combined with GIS methodologies such as remote sensing in dementia research [66]. This technology can provide high-resolution environmental characteristics, and researchers could use this first-hand information to model contextual and environmental risk (e.g., air pollution) and protective factors (e.g., access to publicly open green and blue spaces). However, despite of Web-GIS potential for real-time data management, query, and visualization, it is surprising that this review has not found examples of web-based GIS applications in dementia, when there are successful examples of its use for other chronic diseases, such as cardiovascular [139], diabetes [140], or cancer [141].

Most of the studies were conducted in the United States, followed by UK, Germany and Canada. The low level of publications in other European countries is striking, given the fact that dementia is a top priority in this world region [142]. Unlike multiple sclerosis, which has the most studies in European countries [33], there is a significant gap in GIS/GPS studies in dementia research and care in these countries. We suggest doing more research in this area in future studies. Also, poor service availability and accessibility to dementia-related medical services can negatively affect patients’ life quality [64, 93]. So, more research on resource allocation and policy planning is needed in all countries.

The strength of the current research work is that comprehensive search terms were developed to ensure no article relevant to GIS/GPS and dementia has been missed. Additionally, we applied our search terms to three main databases (PubMed, Web of Science and Scopus) in a health context to retrieve the related papers. Further, the research team had four GIS experts, enabling us to review the articles and properly apply inclusion criteria precisely. However, limiting our search strategy to English articles might not provide a whole picture of the role of GIS/GPS in dementia research.

## Conclusions

Six main applications of GIS and GPS were revealed in this study. Most studies focused on thematic mapping and GPS in the dementia field. Even though the applications of GIS/GPS methodologies and data collection tools in dementia care and research are growing over the past decade, the low number of publications in the European countries is striking. Thus, more analyses using GIS/GPS technologies in European nations are recommended. There is a significant gap in the utilization of GIS/GPS in service planning and dementia care, particularly in resource allocation. Therefore,  more research on resource allocation and policy planning is needed in all countries.

## Supplementary information

**Additional file 1**. PRISMA-ScR-Checklist.

**Additional file 2**. Search Strategy.

**Additional file 3**. Extraction Table.

**Additional file 4**. Techniques.

## Data Availability

All relevant data are included in the manuscript.

## Abbreviations

GIS: Geographic Information Systems
GPS: Global Positioning Systems
