# Supplementary material for: The role of geographic information system and global positioning system in dementia care and research: a scoping review
Source: Int J Health Geogr. 2022 Aug 4;21:8. doi: 10.1186/s12942-022-00308-1 (PMC9354285; doi:10.1186/s12942-022-00308-1)
Supplement: Supplementary file 2 — Additional file 2. Search Strategy. [file 12942_2022_308_MOESM2_ESM.docx]

| **PubMed search strategy**  **Date: 2022-06-06**  **Result: 871** | ("Neurocognitive Disorder"[Title/Abstract] OR "cognitive impairment*"[Title/Abstract] OR "Cognitive Decline"[Title/Abstract] OR "cognitive dysfunction"[Title/Abstract] OR "cognitive dysfunction"[MeSH Major Topic] OR "Mental Deterioration"[Title/Abstract] OR "alzheimer disease"[MeSH Major Topic] OR "alzheimer disease"[Title/Abstract] OR "Alzheimer Dementia"[Title/Abstract] OR "alzheimer s disease*"[Title/Abstract] OR "Dementia"[MeSH Major Topic] OR "Tauopathies"[Title] OR "amentia"[Title] OR "Alzheimer"[Title] OR "Dementia"[Title] OR "wandering"[Title/Abstract] OR "getting lost"[Title/Abstract] OR "patient tracking"[Title/Abstract])  AND  ("geographic information system*"[Title/Abstract] OR "geographical information system*"[Title/Abstract] OR "Geographic Information Systems"[MeSH Major Topic] OR "global positioning system*"[Title/Abstract] OR "GPS"[Title/Abstract] OR "Satellite Imagery"[Title/Abstract] OR "Satellite Imagery"[MeSH Major Topic] OR "Remote Sensing Technology"[MeSH Major Topic] OR "Remote Sensing"[Title/Abstract] OR "choropleth map*"[Title/Abstract] OR "heat map*"[Title/Abstract] OR "dasymetric map*"[Title/Abstract] OR "Spatio-Temporal Analysis"[MeSH Major Topic] OR "Spatio-Temporal Analysis"[Title/Abstract] OR "Spatial Analysis"[Title/Abstract] OR "Spatial Analysis"[MeSH Major Topic] OR "Spatial Autocorrelation"[Title/Abstract] OR "geographic cartography"[Title/Abstract] OR "georeference*"[Title/Abstract] OR "spatial regression"[Title/Abstract] OR "spatial regression"[MeSH Major Topic] OR "Spatial inequality"[Title/Abstract] OR "Geographical inequality"[Title/Abstract] OR "Geographic inequality"[Title/Abstract] OR "Spatial Dependency"[Title/Abstract] OR "Geographic Dependency"[Title/Abstract] OR "Geographically Weighted Regression"[Title/Abstract] OR "Space-Time Geography"[Title/Abstract] OR "Spatial Temporal Analysis"[Title/Abstract] OR "Spatiotemporal Analysis"[Title/Abstract] OR "GIS"[Title] OR "geographical analysis"[Title/Abstract] OR "geographic analysis"[Title/Abstract] OR "spatial access*"[Title/Abstract] OR "geographic access*"[Title/Abstract] OR "geographical access*"[Title/Abstract] OR "geospatial"[Title/Abstract] OR "geographic*"[Title] OR "Disease Hotspot"[MeSH Major Topic] OR "Hotspot analysis"[Title/Abstract] OR "hot spot analysis"[Title/Abstract] OR "Hot-spot analysis"[Title/Abstract] OR "spatial cluster*"[Title/Abstract] OR "geographic cluster*"[Title/Abstract] OR "geographical cluster*"[Title/Abstract] OR "geographic map*"[Title/Abstract] OR "geographical map*"[Title/Abstract] OR "Geographic Mapping"[MeSH Major Topic] OR "geocod*"[Title/Abstract] OR "global system for mobile communications"[Title/Abstract] OR "GSM"[Title]) |
| --- | --- |
| **Scopus search strategy**  **Date: 2022-06-06**  **Result: 1707** | ( ( TITLE-ABS-KEY ( "Neurocognitive Disorder" OR "cognitive impairment*" OR "Cognitive Decline" OR "cognitive dysfunction" OR "Mental Deterioration" OR "alzheimer disease" OR "Alzheimer Dementia" OR "Alzheimer's Disease*" OR "wandering" OR "getting lost" OR "patient tracking" ) ) OR ( TITLE ( "Tauopathies" OR "amentia" OR "Alzheimer" OR "Dementia" ) ) ) AND ( ( TITLE-ABS-KEY ( "geographic information system*" OR "geographical information system*" OR "global positioning system*" OR "GPS" OR "Satellite Imagery" OR "Remote Sensing" OR "choropleth map*" OR "heat map*" OR "dasymetric map*" OR "Spatio-Temporal Analysis" OR "Spatial Analysis" OR "Spatial Autocorrelation" OR "geographic cartography" OR "georeference*" OR "spatial regression" OR "Spatial inequality" OR "Geographical inequality" OR "Geographic inequality" OR "Spatial Dependency" OR "Geographic Dependency" OR "Geographically Weighted Regression" OR "Space-Time Geography" OR "Spatial Temporal Analysis" OR "Spatiotemporal Analysis" OR "geographical analysis" OR "geographic analysis" OR "spatial access*" OR "geographic access*" OR "geographical access*" OR "geospatial" OR "Hotspot analysis" OR "hot spot analysis" OR "Hot-spot analysis" OR "spatial cluster*" OR "geographic cluster*" OR "geographical cluster*" OR "geographic map*" OR "geographical map*" OR "geocod*" OR "global system for mobile communications" ) ) OR ( TITLE ( "GIS" OR "geographic*" OR "GSM") ) ) ) |
| **Web of Sciences search strategy**  **Date: 2022-06-06**  **Result: 1089** | # 1 [[304,430](https://www2.wosgs.ir/wos/woscc/summary/a6b10822-8585-4ad7-a7ee-bdfa14eedd6b-3cc7532a/relevance/1)](https://www.webofscience.com/wos/woscc/summary/a95483c4-f989-498c-8d3c-9ef1632dfaf8-3c6f90f7/relevance/1)  TOPIC: **(("Neurocognitive Disorder" OR "cognitive impairment*" OR "Cognitive Decline" OR "cognitive dysfunction" OR "Mental Deterioration" OR "alzheimer disease" OR "Alzheimer Dementia" OR "Alzheimer's Disease*" OR "wandering" OR "getting lost" OR "patient tracking"))**  Indexes=SCI-EXPANDED, SSCI, A&HCI, CPCI-S, CPCI-SSH, BKCI-S, BKCI-SSH, ESCI Timespan=All years  # 2 [160,007](https://www2.wosgs.ir/wos/woscc/summary/609ec727-b56e-4909-9670-9cb2d9b43c4c-3cc781ff/relevance/1)  TITLE: **(("Tauopathies" OR "amentia" OR "Alzheimer" OR "Dementia"))**Indexes=SCI-EXPANDED, SSCI, A&HCI, CPCI-S, CPCI-SSH, BKCI-S, BKCI-SSH, ESCI Timespan=All years  # 3 [356,675](https://www2.wosgs.ir/wos/woscc/summary/e4d91143-bf18-4d96-98a6-a36ec5c77415-3cc7a055/relevance/1)  #2 OR #1  Indexes=SCI-EXPANDED, SSCI, A&HCI, CPCI-S, CPCI-SSH, BKCI-S, BKCI-SSH, ESCI Timespan=All years  # 4 [381,006](https://www2.wosgs.ir/wos/woscc/summary/ee20e0e0-1b69-4a31-8339-83f90e6848e6-3cc7b87f/relevance/1)  TOPIC: **(("geographic information system*" OR "geographical information system*" OR "global positioning system*" OR "GPS" OR "Satellite Imagery" OR "Remote Sensing" OR "choropleth map*" OR "heat map*" OR "dasymetric map*" OR "Spatio-Temporal Analysis" OR "Spatial Analysis" OR "Spatial Autocorrelation" OR "geographic cartography" OR "georeference*" OR "spatial regression" OR "Spatial inequality" OR "Geographical inequality" OR "Geographic inequality" OR "Spatial Dependency" OR "Geographic Dependency" OR "Geographically Weighted Regression" OR "Space-Time Geography" OR "Spatial Temporal Analysis" OR "Spatiotemporal Analysis" OR "geographical analysis" OR "geographic analysis" OR "spatial access*" OR "geographic access*" OR "geographical access*" OR "geospatial" OR "Hotspot analysis" OR "hot spot analysis" OR "Hot-spot analysis" OR "spatial cluster*" OR "geographic cluster*" OR "geographical cluster*" OR "geographic map*" OR "geographical map*" OR "geocod*" OR "global system for mobile communications") )**  Indexes=SCI-EXPANDED, SSCI, A&HCI, CPCI-S, CPCI-SSH, BKCI-S, BKCI-SSH, ESCI Timespan=All years  # 5 [[87,921](https://www2.wosgs.ir/wos/woscc/summary/7cd8c9b9-7222-4dd5-bf42-ee2ed5c68320-3cc7e126/relevance/1)](https://www.webofscience.com/wos/woscc/summary/8bcae7e9-6913-4866-85b6-927f7a013141-3c706618/relevance/1)  TITLE: **(("GIS" OR "geographic*" OR "GSM"))**  Indexes=SCI-EXPANDED, SSCI, A&HCI, CPCI-S, CPCI-SSH, BKCI-S, BKCI-SSH, ESCI Timespan=All years  # 6 [442,995](https://www2.wosgs.ir/wos/woscc/summary/7b1ebc19-d5f2-40b7-bd1a-1f40982f7b17-3cc80e89/relevance/1)  #5 OR #4  Indexes=SCI-EXPANDED, SSCI, A&HCI, CPCI-S, CPCI-SSH, BKCI-S, BKCI-SSH, ESCI Timespan=All years  # 7 [[1,089](https://www2.wosgs.ir/wos/woscc/summary/c404055e-8891-4fce-8911-3ae6dc38995d-3cc81df4/relevance/1)](https://www.webofscience.com/wos/woscc/summary/05e4e72d-7b24-496b-8fb1-60f10e9a0589-3c706f0e/relevance/1)  #6 AND #3  Indexes=SCI-EXPANDED, SSCI, A&HCI, CPCI-S, CPCI-SSH, BKCI-S, BKCI-SSH, ESCI Timespan=All years |
