# Supplementary material for: The role of geographic information system and global positioning system in dementia care and research: a scoping review
Source: Int J Health Geogr. 2022 Aug 4;21:8. doi: 10.1186/s12942-022-00308-1 (PMC9354285; doi:10.1186/s12942-022-00308-1)
Supplement: Supplementary file 3 — Additional file 3. Extraction Table. [file 12942_2022_308_MOESM3_ESM.docx]

| **ID** | **Title** | **Author(s)** | **Year of**  **publication** | **Study location (Country)** | **Study populations** | **Outcome measures** | **Intervention type** | **Geographical level of analysis (Scale)** | **Aims of the study** | **Important results (GIS related key finding)** | **GIS methodologies** | | | | | **Geospatial** **techniques** | **GIS applications** |
| --- | --- | --- | --- | --- | --- | --- | --- | --- | --- | --- | --- | --- | --- | --- | --- | --- | --- |
|  |  |  |  |  |  |  |  |  |  |  | **Thematic mapping** | **Spatial modelling/ spatial analysis** | **Web-GIS** | **GIS/GPS tools /Technology** | **Space time clustering** |  |  |
| 1 | The Geographic Distribution of Dementia Mortality: Elevated Mortality Rates for Black and White Americans By Place of Birth | M. Maria Glymour, Anna Kosheleva, Virginia G. Wadley, Christopher  Weiss, and Jennifer J. Manly, | 2011 | USA | US-born African-Americans and whites aged 65–89 | Dementia mortality rate | N/A | State | To compare the geographic patterns in all-cause dementia and Alzheimer’s disease mortality rates to those for stroke | Dementia mortality rates follow geographic patterns similar to stroke mortality, with elevated rates among those born in the Stroke Belt. This suggests important roles for geographically patterned childhood exposures in stablishing cognitive reserve. | * | * |  |  |  | Mapping - Empirical Bayes | Disease mapping/  Surveillance (2) |
| 2 | Association of Neighborhood Context, Cognitive Decline, and Cortical Change in an Unimpaired Cohort | Jack F.V. Hunt, Nicholas M. Vogt, Erin M. Jonaitis, William  R. Buckingham, Rebecca L. Koscik, Megan Zuelsdorff1, Lindsay R.  Clark, Carey E Gleason, Menggang Yu, Ozioma Okonkwo, Sterling C. Johnson, Sanjay Asthana, Barbara B. Bendlin, Amy  J.H. Kind | 2021 | USA | 601 cognitively unimpaired participants in the Wisconsin Registry for Alzheimer’s Prevention study and the Wisconsin Alzheimer’s Disease Research Center clinical cohort | Cognitive decline and cortical change | N/A | Census block | To test the hypothesis that neighborhood-level disadvantage is associated with longitudinal measures of neurodegeneration and cognitive decline in an unimpaired cohort | Living in the most highly disadvantaged neighborhoods was associated with accelerated degeneration in Alzheimer’s signature regions and cognitive decline over time. |  | * |  |  |  | OLS^1^ regression models | Risk factor/ Environmental/ Context analysis |
| 3 | Alzheimers Disease: Preliminary Study of Spatial Distribution at Birth Place | H. Jean, J-F. Emard, J.-P. Thouez, L. Houde, Y. Robitaille, r  J. Mathieu, C. Boily, N. Daoud,  M. Beaudry, A.  Cholettef,  R. Bouchard,  F. Veilleux  and D. Gauvreau | 1996 | Canada | A sample of 235 AD cases born in a defined region of Quebec (Canada), between 1895 and 1935 | Geographical  distribution of AD cases | N/A | municipalities classified as rural and urban | To test the hypothesis that the etiology of AD^2^ might be influenced by the environmental factors acting at, or around the place and time of birth | Results showed a statistically significant excess of AD cases in the rural area, an excess of female AD cases in both the rural and urban areas and only the urban area for men. | * |  |  |  |  | Mapping | Disease mapping/  Surveillance |
| 4 | Daily Mood and Out-of-Home Mobility in Older Adults: Does Cognitive Impairment Matter? | Roman Kaspar, Frank Oswald,  Hans-Werner Wahl, Elke Voss,  and Markus Wettstein | 2012 | Germany | 16 persons with  early stage AD, 30 persons with MCI, and 95 cognitively healthy persons | Daily mood and out-of home mobility | GPS tracking | Individual | To relate day-to-day out-of-home mobility to daily mood in a group of community-dwelling older adults with different levels of cognitive functioning | Cognitive status in old age appears to impact on mobility and mood rather than on the mood and out-of-home behavior connection. GPS tracking applications that indicate a potential need to give support to people who get lost out-of-home may help to obviate premature restrictions in mobility in persons suffering from cognitive decline. |  |  |  | * |  | GPS | Basic research |
| 5 | Association between air pollution and risk of vascular dementia: A multipollutant analysis in Taiwan | Chung-Yi Li, Chien-Hsin Li, Santi Martini, Wen-Hsuan Hou | 2019 | Taiwan | 831 adults aged>65 years with vascular dementia,  3324 controls | Risk of vascular dementia | N/A | City/ township | To measure the association between air pollution and risk of vascular dementia | Higher NO2 exposure levels in 3, 5, and 7 years before VaD^3^ diagnosis were significantly and positively associated with an increased risk of VaD. Although less consistent, higher exposure to CO, especially in 5 or 7 years before VaD diagnosis, significantly increased VaD risk. |  | * |  |  |  | Spatial interpolation | Data preparation |
| 6 | Trends of hospitalizations among patients with both cancer and dementia diagnoses in New York 2007-2017 | Bian Liu, Katherine A. Ornstein, Naomi Alpert, Rebecca M. Schwartz, Kavita V. Dharmarajan, Amy S. Kelley, Emanuela Taioli | 2021 | USA | All hospital admissions for patients aged ≥50 years with both cancer and dementia diagnoses (2007-2017). | Hospitalizations among patients who had any diagnosis codes indicative of the presence of both cancer and dementia during the same admission. | N/A | Zip Code Tabulation Areas | To examine hospital admission rates for patients with both cancer and dementia across New York State during 2007-2017 and how these changes are affected by area level socioeconomic factors | An increase over time in the proportion of hospitalized patients with both cancer and dementia diagnosis and its spatial distribution varied with area-level socioeconomic status. Hospitalizations among those with both cancer and dementia diagnoses were associated with a higher socioeconomic status. | * |  |  |  | * | Mapping,  Spatio-temporal Bayesian modelling | Disease mapping/  Surveillance (2) |
| 7 | Fine Particulate Matter and Incident Cognitive Impairment in the REasons for Geographic and Racial Differences in Stroke (REGARDS) Cohort | Matthew Shane Loop, Shia T Kent, Mohammad Z Al-Hamdan, William L Crosson, Sue M Estes, Maurice G Estes Jr, Dale A Quattrochi, Sarah N Hemmings, Virginia G Wadley, Leslie A McClure | 2013 | USA | 3,714 participants Data from the REasons for Geographi and Racial Differences in Stroke cohort | Incident of cognitive impairment | N/A | Residential addresses were linked to 10km PM 2.5 raster grids | To investigate the relationship between fine particulate matter (PM2.5) and incident cognitive impairment in a large biracial cohort of men and women spread across urban and rural regions of the coterminous U.S. The effect of urbanicity of residential address on this relationship was further examined. | Evidence was lacking that the effect of PM2.5 on incident cognitive impairment is robust in a heterogeneous US cohort, even in urban areas. |  | * |  | * |  | Remote sensing & imaging + spatial interpolation | Data preparation |
| 8 | Geographical relation between Alzheimer’s disease and aluminum in drinking water | C. N. Martyn, C. Osmond, J. A. Edwardson, D. J. P. Barker, E. C. Harris, R. F. Lacey | 1989 | United Kingdom | 1185 patients with one of the four categories of dementia | Risk of Alzheimer's disease | N/A | County | To examine the possible relation between exposure to aluminum in water and the development of Alzheimer’s disease | The risk of Alzheimer’s disease was 1.5 times higher in districts where the mean aluminum concentration exceeded 0.11 mg/l than other districts. There was no evidence of a relation between other causes of dementia, or epilepsy, and aluminum concentrations in water. |  | * |  |  |  | Distance based analysis | Data preparation |
| 9 | The Use of Tracking Technologies for the Analysis of Outdoor Mobility in the Face of Dementia: First Steps into a Project and Some Illustrative Findings From Germany | Oswald, Frank  Wahl, Hans Werner  Voss, Elke  Schilling, Oliver  Freytag, Tim  Auslander, Gail  Shoval, Noam  Heinik, Jeremia  Landau, Ruth | 2010 | Germany | 7 healthy elders, 6 individuals with MCI, and 6  individuals with mild dementia | Cognitive functioning, mobility behavior, and well-being | GPS/GIS technology | Individual | To assess out-of-home mobility patterns by means of GPS/GIS technology, to analyze the relationships between cognitive functioning, mobility behavior, and well-being, and to assess the potential of tracking technologies in the diagnosis of various types of cognitive impairment. | The preliminary findings revealed that healthy participants have better health and higher levels of well-being and smaller networks compared to elders who are cognitively impaired. |  |  |  | * |  | GPS | Basic research |
| 10 | Cognitive Decline, Mortality, and Organophosphorus Exposure in Aging Mexican Americans | Paul, Kimberly C.  Ling, Chenxiao  Lee, Anne  To, Tu My  Cockburn, Myles  Haan, Mary  Ritz, Beate | 2017 | USA | 430 Older Mexican Americans from the Sacramento Area Latino Study on Aging | Cognitive decline and mortality rate | N/A | Exposure 500m buffer of individual residence | To investigate whether agriculturally based ambient OP^4^ exposure influences 1) the rate of cognitive decline and mortality and 2) whether these associations are mediated through metabolic or inflammatory biomarkers. | Residential proximity to high levels of agricultural OP pesticide applications was associated with faster rates of cognitive decline and an increased risk of death during follow-up. Also, high ambient exposure is associated with an increased risk of clinically defined dementia. | * | * |  |  |  | Mapping,  Buffer analysis | Disease mapping/  Surveillance,  Data preparation |
| 11 | Mapping Elder Mistreatment Cases: Interactions Between Mistreatment, Dementia, Service Utilization, Access to Services, and Disadvantage | Brian K. Payne &Randy R. Gainey | 2009 | USA | 751 adults | Distribution of elder mistreatment | N/A | Neighborhood | 1. How are reports of elder mistreatment distributed across neighborhoods? 2. Are reports of elder mistreatment cases involving Alzheimer’s-dementia clients distributed differently than other cases? 3.Are services for Alzheimer’s-dementia caregivers distributed across cities in a practical way? 4. Does service utilization vary across neighborhoods? 5. Does neighborhood disadvantage affect burden? | Geographic variation in Alzheimer's cases exists. Neighborhood disadvantage is associated with the likelihood of refusal of services. Broader environmental influences are at least indirectly related to the dynamics surrounding elder mistreatment. | * | * |  |  |  | Mapping,  Distance based analysis | Disease mapping/  Surveillance,  Dementia care/Rehabilitation |
| 12 | Geographic availability and accessibility of day care services for people with dementia in Ireland | Pierse, Tom  Keogh, Fiona  O'Shea, Eamon  Cullinan, John | 2020 | Ireland | 2805 individuals with dementia | Availability and accessibility of day care services for PWD | N/A | CHOs^5^ and their subregions – CHNs^6^ | To examine the geographic distribution of day care services for people with dementia relative to potential need | There is significant variation across the country in the existing capacity of day care centres to cater for people with dementia. 18% of people with dementia do not live within 15kms of their nearest day care centre. | * | * |  |  |  | Mapping,  Distance based analysis | Disease mapping/  Surveillance,  Dementia care/Rehabilitation |
| 13 | Association of Neighborhood-Level Disadvantage with Alzheimer Disease Neuropathology | Powell, W. Ryan  Buckingham, William R.  Larson, Jamie L.  Vilen, Leigha  Yu, Menggang  Salamat, M. Shahriar  Bendlin, Barbara B.  Rissman, Robert A.  Kind, Amy J.H. | 2020 | USA | 447 decedents who donated their brains to 1 of 2 Alzheimer disease research center brain banks | AD Neuropathology rate | N/A | Census block group areas | To establish the feasibility of linking neuropathology data to social determinants of health exposures using neighborhood disadvantage metrics (the validated Area Deprivation Index) and to evaluate the association between neighborhood disadvantage and Alzheimer disease–related neuropathology. | Living in the most disadvantaged neighborhood decile was associated with a 2.18 increased odds of Alzheimer disease neuropathology | * | * |  |  |  | Mapping,  Distance based analysis by geosimulation modelling | Disease mapping/  Surveillance,  Dementia care/Rehabilitation |
| 14 | Hidden Work and the Challenges of Scalability and Sustainability in Ambulatory Assisted Living | Procter, Rob  Wherton, Joe  Greenhalgh, Trisha | 2018 | United Kingdom | 5 cases with complex multi-morbidity (all had both cognitive and physical impairment) | Hidden Work and the Challenges of Scalability and Sustainability | GPS tracking | Individual | Three aims: to understand such individuals’ lived experience of GPS tracking; to facilitate the customization and adaptation of technologies and care services to provide effective, ongoing support; and to explore the possibilities for a co-production methodology that involves patients, families and technologists. | Articulation work performed by professional and lay carers alike to keep GPS tracking services in “working order” has significant implications for their scalability and sustainability and for assisted living services |  |  |  | * |  | GPS | Dementia care/Rehabilitation |
| 15 | Geospatial analysis of environmental risk factors for missing dementia patients | Puthusseryppady, Vaisakh  Coughlan, Gillian  Patel, Martyn  Hornberger, Michael | 2019 | United Kingdom | 210 missing dementia patient case records provided by the police | Missing dementia patient incident | N/A | Lower Super Output Areas | To explore whether there were any hotspot regions of missing incidents and the relationship between outdoor landmark density and missing incidents | Missing patients with dementia are not more prone to getting lost in certain regions compared to others. Missing incidents are a significantly greater problem for patients living at home compared to those in care facilities. Increased presence of outdoor landmarks is an environmental risk factor contributing to patients getting lost, regardless of location. | * | * |  |  | * | Mapping,  (Spatial buffer analysis + OLS),  Global Moran, Local Moran | Disease mapping/  Surveillance (2),  Risk factor/ Environmental/ Context analysis |
| 16 | Geographic disparities in mortality from Alzheimer's disease and related dementias | Akushevich, Igor  Yashkin, Arseniy P.  Yashin, Anatoliy I.  Kravchenko, Julia | 2021 | USA | Deceased US residents, 1999–2018 | Region-specific age-adjusted mortality rates and group-specific rate | N/A | state/county | To provide a detailed state/county-level description of the variation in AD mortality rates across the United States, identify regions that demonstrate exceptionally high or low AD mortality rates, and assess whether the presence of these regions is stable over the 1999–2020 period. To explore the differences between two regions characterized by the highest and lowest U.S.-wide AD mortality rate. | Clusters with the highest and lowest AD were present. These patterns were stable over the 1999–2018 period. circulatory diseases were the primary contributors to the differences. Furthermore, differences in mortality associated with race/ethnicity, location of death, and place of residence were a major factor in describing differences in both total and disease-group-related mortality. | * |  |  |  |  | Mapping | Disease mapping/  Surveillance |
| 17 | Spatial distribution of deaths due to Alzheimer’s disease in the state of São Paulo, Brazil | Almeida, Milena Cristina da Silva  Gomes, Camila de Moraes Santos  Nascimento, Luiz Fernando Costa | 2014 | Brazil | 645 municipalities in the state of São Paulo, Brazil, between 2004 and 2009 | AD mortality rate | N/A | State | To identify spatial distribution patterns of mortality due to Alzheimer’s disease in the state of São Paulo. | The results identified municipalities with high rates of deaths due to AD that merit intervention. | * |  |  |  | * | Mapping,  Global Moran, Local Moran | Disease mapping/  Surveillance (2) |
| 18 | Geographic Clusters of Alzheimer’s Disease Mortality Rates in the USA: 2008-2012 | Amin, R. W.  Yacko, E. M.  Guttmann, R. P. | 2018 | USA | All counties in the contiguous United States, for all years 2008-2012 from the Wonder Multiple Cause of Death Database | AD mortality rate | N/A | state/county | To investigate geographic clusters of high and low AD-related mortality across the contiguous United States. | Three large clusters had elevated age-adjusted AD mortality of at least 60% above the national average. | * |  |  |  | * | Mapping,  spatial scan statistic | Disease mapping/  Surveillance (2) |
| 19 | The Impact of Built and Social Environmental Characteristics on Diagnosed and Estimated Future Risk of Dementia | Bagheri, Nasser  Mavoa, Suzanne  Tabatabaei-Jafari, Hossein  Knibbs, Luke D  Coffee, Neil T  Salvador-Carulla, Luis  Anstey, Kaarin J | 2021 | Australia | 25,511 patients aged 65 years and older | Dementia risk | N/A | SA1^7^ | 1)To assess the associations between built and social environments’ characteristics (i.e., public open spaces, air pollution, density of busy roads, walkability, and social fragmentation) with diagnosed cases of dementia and the estimated dementia risk in a primary health care setting using general practice clinical data; 2) To assess spatial variation in dementia risk and diagnosed cases of dementia is investigated across neighborhoods (SA1s) in the study area. | The results identify a spatial heterogeneity pattern in the estimated risk of dementia and for diagnosed cases of dementia in primary care setting in the study areas. | * | * |  |  | * | Mapping,  Local Moran,  Land use regression | Disease mapping/  Surveillance (2),  Data preparation |
| 20 | Outdoor life in dementia: How predictable are people with dementia in their mobility? | Bayat, Sayeh  Mihailidis, Alex | 2021 | Canada | 7 older adults with dementia and 8 healthy older adults | Predict the participant's future destinations | GPS tracking devices | Individual | To explore the extent to predict future whereabouts of PWD^8^ by learning from their past mobility patterns using GPS tracking devices | 4-week record of mobility patterns displays 95% potential predictability of PWD destinations. |  |  |  | * |  | GPS | Basic research |
| 21 | Anomaly Detection to Increase Commuter Safety for Individuals with Cognitive Impairments | Chang, Yao Jen  Wang, Frank Tsen Yung  Chen, Shu Fang  Ma, Tien Shyan | 2011 | Taiwan | 4 adults with cognitive impairments | Real-time anomaly for  traveling individuals such as taking the wrong bus, missing a bus stop, or getting off at the wrong stop. | A commercial off-the-shelf PDA with built-in GPS | Individual | To assess the possibility of using handheld devices to increase commuter safety for adults with cognitive impairments | The data show that participants’ awareness of anomalies significantly increased in target response, thus improving trip safety during intervention phases. |  |  |  | * |  | GPS | Dementia care/Rehabilitation |
| 22 | Geographic Variation in Hospice Use in the United States in 2002 | Connor, Stephen R.  Elwert, Felix  Spence, Carol  Christakis, Nicholas A. | 2007 | USA | Population data from the Standard Analytic File Hospice maintained by the CMS and from the Compressed Mortality File maintained by the National Center for Health Statistics | Hospice Utilization Ratio | N/A | state/county | To describe the whole population of hospice users, and nonusers, in the United States. | The study finds considerable geographic differences in hospice utilization. | * |  |  |  |  | Mapping | Disease mapping/  Surveillance |
| 23 | Geographical distribution of Alzheimer’s disease cases at birth and the geochemical profile of Saguenay-Lac-Saint-Jean/Qui-Be, Canada | Emard, Jean François  Andre, Pierre  Thouez, Jean Pierre  Mathieu, Jean  Boily, Camil  Beaudry, Michel  Cholette, Andree  Robitaille, Yves  Bouchard, Remi  Daoud, Nicolas  Veilleux, Francine  Gauvreau, Denis | 1994 | Canada | AD cases, geochemical profile | AD rate | N/A | municipality | To explore the possible links between the geographical distribution of AD cases according to their birth place and the geochemical profile of the SLSJ^9^ territory in the province of Quebec. | No single geochemical element seems to be associate with the spatial distribution of cases. It is, however, still possible that a certain synergistic effect between two or several elements could be implied in the development of the disease. | * | * |  |  |  | Mapping,  Spatial interpolation | Disease mapping/  Surveillance, Data preparation |
| 24 | Geographical Differences in the Occurrence of Alzheimer’s Disease Mortality: United States Versus Puerto Rico | Figueroa, Raul  Steenland, Kyle  MacNeil, Jessica R  Levey, Allan I  Vega, Irving E | 2008 | USA and Puerto Rico | Population data per region for the United States and Territories | AD mortality rate | N/A | Region | To investigate the occurrence of AD mortality from 1999 to 2004 in Puerto Rico, and compare the mortality rates to those in the United States. | The results show an increasing trend in Alzheimer’s disease mortality rate in both the United States and Puerto Rico. | * |  |  |  |  | Mapping | Disease mapping/  Surveillance |
| 25 | No geographic correlation between Lyme disease and death due to 4 neurodegenerative disorders, United States, 2001–2010 | Forrester, Joseph D.  Kugeler, Kiersten J.  Perea, Anna E.  Pastula, Daniel M.  Mead, Paul S. | 2015 | USA | 256,373 Confirmed Lyme disease cases | Lyme disease incidence rates, death rates for Alzheimer disease, ALS, MS, and Parkinson disease | N/A | state | To compare the distribution of Lyme disease cases in the United States with the distributions of deaths due to AD, ALS^10^, MS^11^, and Parkinson disease | No geographic correlations were identified in comparing disease distribution. An inverse correlation was detected between Lyme disease and Alzheimer disease. | * |  |  |  | * | Mapping,  Local Moran | Disease mapping/  Surveillance (2) |
| 26 | Ubiquitous Health Management System with Watch-Type Monitoring Device for Dementia Patients | Shin, Dongkyoo  Shin, Dongmin  Shin, Dongil | 2014 | Korea | 8 dementia patients | Amount of outdoor action | Smart watch with GPS, accelerometer, and illumination sensor, | Individual | To develop a GPS included watch-type device (smart watch) for monitoring health data | The developed ubiquitous health management system for dementia patients not only monitors patients’ locations but also manages patients’ health by determining patients’ activity according to the data derived with the step detection algorithm, along with the ambient light sensor and accelerometer. According to the results of the experiments, normal steps have 96% accuracy in detection and on average showed 94% accuracy. |  |  |  | * |  | GPS | Dementia care/Rehabilitation |
| 27 | Use of the global positioning system to measure the out-of-home mobility of older adults with differing cognitive functioning | Shoval, Noam  Wahl, Hans Werner  Auslander, Gail  Isaacson, Michal  Oswald, Frank  Edry, Tamar  Landau, Ruth  Heinik, Jeremia | 2011 | Israel | 41 participants in the SenTra (that is studying the outdoor activities of elderly people using advanced tracking technologies) project | Out-of-home mobility of older adults with differing cognitive functioning | GPS | Individual | does the timing and distance of out-of-home mobility vary with the level of cognitive impairment? | The spatial range of the mobility of elderly people with cognitive impairment is severely restricted, with most out-of- home time spent in close proximity to their residences. it’s been concluded that GPS is an advanced research tool able to understand out-of-home behavior better than was possible with previous methods. |  |  |  | * |  | GPS | Basic research |
| 28 | Distance to screening site and older adults’ participation in cognitive impairment screening | Harada, Kazuhiro  Lee, Sangyoon  Shimada, Hiroyuki  Lee, Sungchul  Bae, Seongryu  Anan, Yuya  Harada, Kenji  Suzuki, Takao | 2017 | Japan | 9616 respondents of subcohort data from the National Center for Geriatrics and Gerontology-Study of Geriatric Syndrome | Distance to the screening site | N/A | Individuals | To examine whether a shorter distance to the screening site predicted participation in screening for cognitive impairment, and whether interactive effects of the distance and psychological factors on the participation would be observed among community dwelling older adults. | A shorter distance to a screening site predicted participation in screening for cognitive impairments among community dwelling older adults regardless of their psychological status. For the prevention or delay of dementia, it is important to promote participation in screening for cognitive impairment. |  | * |  |  |  | Distance based analysis | Dementia care/Rehabilitation |
| 29 | Mortality rate of Alzheimer’s disease in Japan: secular trends, marital status, and geographical variations | Imaizumi, Yoko | 1992 | Japan | AD patients | Mortality rate of AD | N/A | Prefectures | To analyze the changes and the geographical variations in the AD death rate during the period from 1979 to 1990 in Japan. | Geographical variation of AD death rate in each prefecture during the period from 1979 to 1990. | * |  |  |  |  | Mapping | Disease mapping/  Surveillance |
| 30 | Compliance and data quality in GPS-based studies | Isaacson, Citation  Shoval, Noam  Wahl, Hans-Werner  Oswald, Frank  Auslander, Gail | 2014 | Israel | 89 men and women, 63–92 years old (53 cognitively impaired, 39 healthy) | Validity of the GPS data collected, Mobility | GPS | Individual | To present a method that combines the use of a GPS receiver with RFID^12^ technology that was implemented in research on time–space activities of elderly persons with cognitive impairment. | The feasibility of a method with which it is possible to measure compliance rates in GPS based studies. |  |  |  | * |  | GPS | Basic research |
| 31 | Geographical Variation in Opioid Use in Elderly Patients with Dementia: A Nationwide Study | Jensen-Dahm, Christina  Zakarias, Johanne Købstrup  Gasse, Christiane  Waldemar, Gunhild | 2019 | Denmark | Elderly (≥65 years) population of 98 Denmark municipalities  with (n = 36,014) and without dementia (n = 1,011,787) in 2015 | Prevalence of opioid use | N/A | Municipalities | To investigate potential geographical variation in opioid use among elderly with and without dementia. | In the entire elderly population of Denmark, it was found pronounced geographical variation in opioid use, which was not explained by differences in age, sex, and comorbidity. | * |  |  |  |  | Mapping | Disease mapping/  Surveillance |
| 32 | Temporal trends in the mortality rate of Alzheimer's disease and other dementias attributable to smoking, 1990–2017 | Jiang, Yanfeng  Man, Qiuhong  Liu, Zhenqiu  Wang, Yingzhe  Suo, Chen  Jin, Li  Dong, Qiang  Cui, Mei  Chen, Xingdong | 2020 | Worldwide | Dementia deaths attributable to smoking were obtained from the Global Burden of Disease Study 2017 | Age-standardized mortality rate | N/A | Countries | To estimate smoking-attributable dementia-related deaths by sex and age group at the global and national levels from 1990 to 2017. | The number of dementia deaths has increased steadily at the global level over the past 27 years, and there were undesirable increases in the dementia age-standardized mortality rates in some countries. | * |  |  |  |  | Mapping | Disease mapping/  Surveillance |
| 33 | Temporal and Geographic Variation in the Incidence of Alzheimer’s Disease Diagnosis in the US between 2007 and 2014 | Kirson, Noam Y.  Meadows, Eric S.  Desai, Urvi  Smith, Brian P.  Cheung, Hoi Ching  Zuckerman, Peter  Matthews, Brandy R. | 2019 | USA | 5% random sample of US Medicare beneficiaries for services provided aged 65 years or older between  1999 and 2014 | Incidence of AD | N/A | Core-based statistical area | To describe the incidence of AD overall and by geographic region, using data from a nationally representative 5% random sample of US Medicare beneficiaries. | There is a considerable geographic variation in diagnosed incidence of AD in the United States. | * |  |  |  |  | Mapping | Disease mapping/  Surveillance |
| 34 | Electronic tracking of patients with dementia and wandering using mobile phone technology | Miskelly, Frank | 2005 | United Kingdom | Seven  males and four females, of whom three were <70 years, two  were 71–80 years and six were >80 years | Locate missing persons | GPS | Individual | To describe the use of GPS technology to locate missing persons anywhere in the country, except inside buildings and on public transport, and to an accuracy of approximately 5 meters. | The provided GPS-enabled mobile phone is the first to successfully locate people with dementia who are lost. |  |  |  | * |  | GPS | Dementia care/Rehabilitation |
| 35 | Long-term exposure to ambient air pollution and risk of dementia: Results of the prospective Three-City Study | Mortamais, Marion  Gutierrez, Laure Anne  de Hoogh, Kees  Chen, Jie  Vienneau, Danielle  Carrière, Isabelle  Letellier, Noémie  Helmer, Catherine  Gabelle, Audrey  Mura, Thibault  Sunyer, Jordi  Benmarhnia, Tarik  Jacquemin, Bénédicte  Berr, Claudine | 2021 | France | 7066 participants aged ≥65 years between 1999 and 2001 and followed for 12 years | Risk of dementia | N/A | Neighborhoods | To investigate the association between long-term exposure to air pollutants and incidence of dementia in older adults using reliable diagnostic tools in a large population-based cohort in France. | Long-term exposure to PM2.5 is associated with all-cause dementia, Alzheimer's disease, and vascular/mixed dementia incidence. |  | * * |  |  |  | Multilevel spatial random-effects Cox proportional hazards model,  land use regression model | Risk factor/ Environmental/ Context analysis,  Data preparation |
| 36 | Environmental Factors Affecting Cognitive Function among Community-Dwelling Older Adults: A Longitudinal Study | Motohiro, Atsushi  Abe, Takafumi  Okuyama, Kenta  Onoda, Keiichi  Ito, Tomoko  Isomura, Minoru  Nabika, Toru  Kumakura, Shunichi | 2021 | Japan | 485 older adults aged ≥60 years | The association between environmental factors and cognitive function | N/A | Census | To examine the factors that contribute to cognitive decline among older adults, with the aim of developing effective measures against its progression. | Neighborhood environment has an effect on cognitive decline. The risk of cognitive decline was higher in more hilly environments (increased elevation and hilliness). |  | * |  |  |  | Buffer analysis | Data preparation |
| 37 | Geographical Variation in Dementia Mortality in Italy, New Zealand, and Chile: The Impact of Latitude, Vitamin D, and Air Pollution | Russ, Tom  Murianni, Laura  Icaza, Gloria  Slachevsky, Andrea  Starr, John | 2016 | Italy, New Zealand, and Chile | Population for 20 regions in Italy, 20 District Health Board areas in New Zealand and 29 Health Service areas in Chile | Standardised mortality ratios for deaths in dementia | N/A | Regions/District/health service areas | To verify if the relative insufficiency of vitamin D related to sunlight exposure due to the geographical latitude contributes to dementia risk, the opposite latitudinal gradient should be observed in southern hemisphere settings. | Increased dementia Standard Mortality Ratios in the north of Italy compared to the south, a possible inverse pattern in New Zealand – higher rates in the south compared to the north – at least in women, but increased standard Mortality Ratios in central and northern Chile. | * |  |  |  |  | Mapping | Disease mapping/  Surveillance |
| 38 | A Virtual Earth Model of the Dementias in China | Robertson, Hamish  Nicholas, Nick  Travaglia, Jo  Hayen, Andrew  Georgiou, Andrew | 2017 | China | Population of all Chinese counties (2872) to visualize  dementia-affected population | Complex nature of population ageing and the dementias | N/A | Counties | To explore one of the options for visualising the complexities of demographic change in equally complex geographic and systemic environments. | The research project adds to that complex emerging picture by modeling and visualising, in a spatial form, the scale of the issues that rapid population ageing presents for China and where its effects will be felt most strongly. | * |  |  |  |  | Mapping | Disease mapping/  Surveillance |
| 39 | Geographical variation in dementia: examining the role of environmental factors in Sweden and Scotland | Russ, Tom C.  Gatz, Margaret  Pedersen, Nancy L.  Hannah, Jean  Wyper, Grant  Batty, G. David  Deary, Ian J.  Starr, John M. | 2015 | Sweden and United Kingdom | Swedish Twin Registry (n=27,680) and the 1932 Scottish Mental Survey cohort (n=37,597). | Dementia rate | N/A | County | To estimate the magnitude of geographical variation in dementia rates and suggest explanations for this variation. | There is geographical variation in dementia rates. Substantial non-random geographical variation in dementia rates in two countries; the general pattern was of higher rates in the north compared with the south. | * | * * |  |  |  | Mapping,  Bayesian disease mapping,  Besag-York-Mollie model | Disease mapping/  Surveillance (3) |
| 40 | A two-decade comparison of prevalence of dementia in individuals aged 65 years and older from three geographical areas of England: results of the Cognitive Function and Ageing Study I and II | Matthews, Fiona E.  Arthur, Antony  Barnes, Linda E.  Bond, John  Jagger, Carol  Robinson, Louise  Brayne, Carol | 2013 | United Kingdom | Population data of Medical Research Council Cognitive Function and Ageing Study in three of the original study areas in England and Wales | Prevalence of dementia | N/A | Postcode | To investigate whether the prevalence of dementia had changed in the past two decades by repeating the same approach and diagnostic methods as used in the MRC CFAS^13^ in three of the original study areas in England. | There is a variation in prevalence of dementia across England, applied to local age structures, which leads to differences in expected prevalence by geography. | * |  |  |  |  | Mapping | Disease mapping/  Surveillance |
| 41 | Amyotrophic lateral sclerosis and parkinsonism dementia on Guam, 1945-1972, Descriptive epidemiology | Reed, Dwayne M.  Brody, Jacob A. | 1975 | USA | 350 Chamorro cases of ALS and 213 cases of parkinsonism-dementia | Rates of ALS and PD diseases, | N/A | - | To describe the changing epidemiologic patterns of occurrence of amyotrophic lateral sclerosis and parkinsonism-dementia during the past 25 years | Geographic differences in the distribution of the diseases were observed | * |  |  |  |  | Mapping | Disease mapping/  Surveillance |
| 42 | Increased Dementia Mortality in West Virginia Counties with Mountaintop Removal Mining? | Salm, A. K.  Benson, Michael J. | 2019 | USA | Population and death certificate data | Dementia and AD Mortality, | N/A | county | To compare county dementia mortality statistics over a period spanning 2001–2015 | satellite imaging data revealed a highly significant positive correlation between the number of distinct mining sites vs. both mean and cumulative vascular and unspecified dementia mortality over the 15-year period. Based on these results, it seems that inhalation of PM associated with mountaintop removal mining contributes to dementia mortality of the vascular or unspecified types. |  |  |  | * |  | Remote sensing and imaging | Risk factor/ Environmental/ Context analysis |
| 43 | Implications of Geographic Information Systems for targeted recruitment of older adults with dementia and their caregivers in the community: A retrospective analysis | Scerpella, Danny L.  Adam, Atif  Marx, Katherine  Gitlin, Laura N. | 2019 | USA | 234 caregivers and persons living with dementia | Geographic trends of PWD | N/A | census tract | To demonstrate the ability of GIS methodologies to leverage geographic trends in recruitment of an older adult population of people living with dementia in the community and their caregivers | This study successfully defined specific geographic regions that overlapped with a large number of known dementia dyad locations obtained via traditional recruitment efforts. | * | * |  |  |  | Mapping,+  Spatial buffer analysis + overlay analysis | Disease mapping/  Surveillance |
| 44 | Real-Time Detection of Spatial Disorientation in Persons with MCI^14^ and Dementia | Schaat, Samer  Koldrack, Philipp  Yordanova, Kristina  Kirste, Thomas  Teipel, Stefan | 2019 | Germany | 13 people with amnestic  MCI or clinically probable Alzheimer’s disease dementia | Real-Time detection of spatial  disorientation | GPS | Individual | To examine the use of accelerometers in cognitively impaired people to detect disoriented behavior in real-time in an urban environment | Accelerometric data are able to capture the uniformity and activity of a person’s walking, which are identified as the most informative locomotion features of spatially disoriented behavior. This serves as an important basis for real-time navigation assistance. |  |  |  | * |  | GPS | Dementia care/Rehabilitation |
| 45 | A geographic approach to measuring and organizing affordable medical and therapeutic tourism for people with dementia | Semenova, Zoya A.  Chistobaev, Anatoliy I.  Dildina, Valeriya P. | 2020 | World wide | 100 countries for medical tourism for people suffering from dementia | Affordable medical and therapeutic tourism | N/A | Country | To cover the geographical features of the organisation of medical tourism for a group of the world’s population suffering from a brain disease – dementia | The map shows country ranking by the level of development of affordable tourism for people suffering from dementia. | * |  |  |  |  | Mapping | Disease mapping/  Surveillance |
| 46 | Exposure to ambient air pollutants and the onset of dementia in Québec, Canada | Smargiassi, Audrey  Sidi, Elhadji Anassour Laouan  Robert, Louis Etienne  Plante, Céline  Haddad, Mona  Gamache, Philippe  Burnett, Rick  Goudreau, Sophie  Liu, Ling  Fournier, Michel  Pelletier, Eric  Yankoty, Ines | 2020 | Canada | Adults aged 65 years and older | The association between exposure to air pollutants, and onset of dementia | N/A | Postal code | To determine the association between the onset of dementia such as AD and associated diseases and exposure to ambient levels of PM2.5, NO2 and distance to major roads. | The onset of dementia may be related to residential exposure to PM2.5, NO2, and distance to major roads. (PM2.5, NO2, distances to major roads (Montreal) were associated with dementia onset.) |  |  |  | * |  | Remote sensing and imaging | Data preparation |
| 47 | Using the concept of activity space to understand the social health of older adults living with memory problems and dementia at home | Sturge, Jodi  Klaassens, Mirjam  Lager, Debbie  Weitkamp, Gerd  Vegter, Daan  Meijering, Louise | 2020 | Netherlands | 7 older adults experiencing memory problems and living at home | Activity space to examine the social health | GPS | Individual | To use the concept of activity space to examine the social health of older adults with memory problems and dementia who live at home. | Findings show that participants interact independently in routine activity spaces but depend on others to participate in occasional activity spaces. Findings from this research can inform social health care planning and contribute to the development of dementia-friendly practices which are based on the perspectives of older adults experiencing memory problems. |  |  |  | * |  | GPS | Dementia care/Rehabilitation |
| 48 | Neighborhood Sidewalk Environment and Incidence of Dementia in Older Japanese Adults: The Japan Gerontological Evaluation Study Cohort | Tani, Yukako  Hanazato, Masamichi  Fujiwara, Takeo  Suzuki, Norimichi  Kondo, Katsunori | 2021 | Japan | 76,053 participants aged 65–103 years with dementia | Incidence of Dementia | N/A | Neighborhoods | To examine the association between neighborhood sidewalk environment and dementia in Japan. | Living in a neighborhood with a high level of sidewalk installation was associated with low dementia incidence in urban areas. (higher sidewalk coverage was associated with lower dementia incidence in urban areas) |  | * |  |  |  | Aerial photograph analysis | Data preparation |
| 49 | Measuring Life Space in Older Adults with Mild-to-Moderate Alzheimer’s Disease Using Mobile Phone GPS | Tung, James Yungjen  Rose, Rhiannon Victoria  Gammada, Emnet  Lam, Isabel  Roy, Eric Alexander  Black, Sandra E.  Poupart, Pascal | 2013 | Canada | Nineteen community-dwelling older adults with mild-to-moderate AD | Validity of Mobile Phone GPS, measuring life space (defined as the geographical area a person covers in daily life) | GPS | Individual | To evaluate the construct validity of a GPS system to provide quantitative measurements of global movement for individuals with mild-to moderate AD | This study demonstrated that GPS-derived area and perimeter: distinguished mild-to-moderate AD patients were strongly correlated with physical function and affective state. These findings confirm the ability of GPS technology to assess life space behavior and may be particularly valuable to continuously monitor functional decline associated with neurodegenerative disease, such as AD. |  |  |  | * |  | GPS | Basic research |
| 50 | Neighborhood Integration and Connectivity Predict Cognitive Performance and Decline | Watts, Amber  Ferdous, Farhana  Moore, Keith Diaz  Burns, Jeffrey M | 2015 | USA | 64 older adults with and without mild AD | Cognitive Performance and Decline | N/A | Neighborhoods | 1)To determine how objective measures of two different neighborhood characteristics, connectivity and integration, are related to cognitive function and decline, 2) To evaluate whether these characteristics influence older adults without dementia and with dementia in different ways. | The results suggest that neighborhood integration and neighborhood connectivity are independently and differentially associated with cognitive performance and decline and that this pattern of results differs between individuals with and without AD. |  | * |  |  |  | space syntax analysis | Data preparation |
| 51 | Caregiving burden and out-of-home mobility of cognitively impaired care-recipients based on GPS tracking | Werner, Shirli  Auslander, Gail K.  Shoval, Noam  Gitlitz, Tamar  Landau, Ruth  Heinik, Jeremia | 2012 | Israel | Seventy-six dyads (care-recipients and caregivers) for a period of four weeks | Out-of-home mobility | GPS | Individual | To clarify the relationship between caregiving burden and out-of-home mobility of care recipients using GPS technology. | Greater caregiving burden was associated with lower cognitive status and poor behavioral/emotional status among the care recipients. |  |  |  | * |  | GPS | Dementia care/Rehabilitation |
| 52 | Behavioral Competence and Emotional Well-Being of Older Adults with Mild Cognitive Impairment; Comparison with Cognitively Healthy Controls and Individuals with Early-Stage Dementia | Wettstein, Markus  Seidl, Ulrich  Wahl, Hans Werner  Shoval, Noam  Heinik, Jeremia | 2014 | Israel and Germany | 257 older adults aged 59 to 91 years with MCI differed from HC and persons AD | Behavioral competence and emotional well-being | GPS tracking kit | Individual | To examine the objective and behavioral competence as well as the emotional well-being of MCI older adults compared to HC^15^ and persons with dementia (AD) individuals | Regarding perceived behavioral competence and emotional well-being, MCI individuals were more similar to the AD group and below the HC group’s mean levels. Findings suggest that a differentiated view of MCI individual’s competence and emotional well-being is in place. |  |  |  | * |  | GPS | Basic research |
| 53 | Cognitive status moderates the relationship between out-of-home behavior, environmental mastery and affect | Wettstein, Markus  Wahl, Hans Werner  Shoval, Noam  Auslander, Gail  Oswald, Frank  Heinik, Jeremia | 2014 | Israel and Germany | 35 persons with early-stage dementia of the Alzheimer's type (DAT), 76 individuals with MCI and 146 cognitively healthy study participants | out-of-home behavior | GPS technology | Individual | 1) To investigate the multidimensionality of OOHB^16^ by using multiple indicators, which were assessed based either on GPS technology or on self-reports. 2) To examine the relationships of different OOHB dimensions with environmental mastery and affective well-being are moderated by cognitive status | Findings point to the possibility that relationships between OOHB and well-being depend on the congruence between available cognitive resources and the complexity of the OOHB dimension considered. |  |  |  | * |  | GPS | Basic research |
| 54 | Identifying Mobility Types in Cognitively Heterogeneous Older Adults Based on GPS-Tracking: What Discriminates Best? | Wettstein, Markus  Wahl, Hans Werner  Shoval, Noam  Auslander, Gail  Oswald, Frank  Heinik, Jeremia | 2015 | Israel and Germany | Cognitively heterogeneous sample of N = 257 older adults | Mobility Types | GPS tracking technology | Individual | To assess and analyze the interindividual heterogeneity of out-of-home mobility using GPS technology in a cognitively heterogeneous group of older adults | Based on cluster analysis, three mobility types (“Mobility restricted,” “Outdoor oriented,” “Walkers”) were identified, which could be predicted based on socio-demographic indicators, activity, health, and cognitive impairment status using discriminant analysis. Particularly demented individuals and persons with worse health exhibited restrictions in mobility. |  |  |  | * |  | GPS | Basic research |
| 55 | Out-of-Home Behavior and Cognitive Impairment in Older Adults: Findings of the SenTra Project | Wettstein, Markus  Wahl, Hans Werner  Shoval, Noam  Oswald, Frank  Voss, Elke  Seidl, Ulrich  Frölich, Lutz  Auslander, Gail  Heinik, Jeremia  Landau, Ruth | 2015 | Israel and Germany | 257 older adults aged 59 to 91 years included 35 persons with early-stage AD, 76 persons with MCI, and 146 cognitively healthy persons of SenTra Project | Out-of-Home Behavior, | GPS tracking device | Individual | To explore differences in the out-of-home behavior of community-dwelling older adults with different cognitive impairments. | Predicting cognitive impairment status by out-of-home behavior and a range of confounders by means of multinomial logistic regression revealed that only cognitively demanding activities showed at least a marginally significant difference between MCI and cognitively CH and were highly significant between AD and CH. |  |  |  | * |  | GPS | Basic research |
| 56 | Epidemiology of Presenile Alzheimer's Disease in Scotland (1974-88) I. Non-Random Geographical Variation | Whalley, L. J.  Thomas, B. M.  McGonigal, G.  McQuade, C. A.  Swingler, R.  Black, R. | 1995 | United Kingdom | 5874 hospital records of the 6581 cases | Incidence rates of ‘probable’ presenile Alzheimer's disease | N/A | Postcode sectors | To examine the role of birth characteristics, occupation and survival in the incidence of AD PSD^17^. | There was non-random geographical distribution of AD PSD. | * |  |  |  |  | Mapping | Disease mapping/  Surveillance |
| 57 | Neighborhood environment and dementia in older people from high-, middle- and low-income countries: results from two population-based cohort studies | Wu, Yu Tzu  Brayne, Carol  Liu, Zhaorui  Huang, Yueqin  Sosa, Ana Luisa  Acosta, Daisy  Prina, Matthew | 2020 | United Kingdom; China; Dominican Republic and Mexico | Based on two population-based cohort studies of people aged≥65: the Cognitive Function and Ageing Study II in UK (N = 4955) and a subset of the 10/66 study in China, Dominican Republic and Mexico (N = 3386). | Dementia rate | N/A | country | To investigate the cross-sectional associations between neighborhood amenities and dementia in older people from high-, middle- and low-income countries. | 1) The different relationships across cohorts may indicate a varying role for local amenities in diverse settings.2) In both cohorts, living far from daily life amenities (post office or convenience store) was associated with higher odds of dementia. 3) A higher availability of local green and blue spaces was not found to be associated with dementia in either cohorts. |  | * |  |  |  | Spatial buffer analysis | Data preparation |
| 58 | Perceived and objective availability of green and blue spaces and quality of life in people with dementia: results from the IDEAL program | Wu, Yu Tzu  Clare, Linda  Jones, Ian Rees  Nelis, Sharon M.  Quinn, Catherine  Martyr, Anthony  Victor, Christina R.  Lamont, Ruth A.  Rippon, Isla  Matthews, Fiona E. | 2021 | United Kingdom | 1540 community-dwelling people with dementia | PWD rate | N/A | - | To investigate the associations between quality of life and both perceived and objective availability of local green and blue spaces in people with dementia, including potential variation across rural/urban settings and those with/without opportunities to go outdoors. | 1) The positive association between perceived availability and quality of life was stronger for urban than rural residents but did not differ between participants with and without opportunities to go outdoors. 2) Only perceived availability was related to quality of life in people with dementia. |  | * |  |  |  | Spatial buffer analysis | Data preparation |
| 59 | Detecting spatiotemporal clusters of dementia mortality in the United States, 2000-2010 | Xu, Wei  Wu, Changshan | 2018 | USA | US multiple cause of death files for 2000- 2010, county populations, and county location data | Dementia mortality | N/A | county | To employee the space-time scan statistic to identify spatiotemporal clusters of dementia mortality in the contiguous United States. | Low risk and high-risk clusters were found in the study area. Improvement in relative risk of Alzheimer's disease/ dementia mortality exists in highly likely clusters over the decade. | * |  |  |  | * | Mapping,  spatial scan statistic | Disease mapping/  Surveillance (2) |
| 60 | Justice in Dementia Care Resource Allocation: How Should We Plan for Dementia Services? | Yen, Chia Feng  Lin, Shyang Woei | 2019 | Taiwan | 9704 PWD living in the community  (Adults with disability 18 years or older ) | Service availability and accessibility | N/A | County/City | To use two newly developed indicators; profit willing distance and tolerance limited distance, to profile the service availability and accessibility of the 22 administrative areas in Taiwan and facilitate justice-based resource allocation by the central government. | The profit willing distance and tolerance limited distance appear to add more value to support the policy‐making process than some traditional indices which focus primarily on supply and demand by examining factors such as user–service ratio, population in need of services, and number of providers. | * | * |  |  |  | Mapping,  Distance based analysis | Disease mapping/  Surveillance, Dementia care/Rehabilitation, Planning |
| 61 | The Effect of the Global Positioning System on the Driving Performance of People with Mild Alzheimer’s Disease | Yi, Jewel  Lee, Hoe Chung Yeung  Parsons, Richard  Falkmer, Torbjorn | 2014 | Australia | Twenty-eight drivers with mild to very mild AD | Driving performance | GPS | GPS | To investigate the effectiveness of the GPS in assisting drivers with mild AD in finding their destination safely. | using single, simple auditory instructions with the absence of the visual output of the GPS could potentially help people with mild AD to improve their driving ability and reach their destination. |  |  |  | * |  | GPS | Dementia care/Rehabilitation |
| 62 | Temporal Trends and Geographic Variations in Dementia Mortality in China Between 2006 and 2012 Multilevel Evidence from a Nationally Representative Sample | Yin, Peng  Feng, Xiaoqi  Astell-Burt, Thomas  Page, Andrew  Liu, Jiangmei  Liu, Yunning  Liu, Shiwei  Wang, Lijun  Wang, Limin  Zhou, Maigeng | 2016 | China | Data of 161 counties and districts from the nationally representative China Mortality Surveillance System | Dementia mortality | N/A | Region | To explore the temporal trends and geographic variations in dementia mortality in China | 1) Mortality rates increased in rural areas and decreased in urban areas during 2006 to 2012. 2) regional inequalities in dementia mortality are salient, and the increase in mortality rates in rural areas is an emerging public health challenge in China. Tailored preventive health strategies should be in place to narrow down this avoidable and wholly unnecessary inequality. | * | * |  |  |  | Mapping,  some spatial analysis | Disease mapping/  Surveillance,  Data preparation |
| 63 | Traffic-related Noise Exposure and Late-life Dementia and Cognitive Impairment in Mexican–Americans | Yu, Yu  Mayeda, Elizabeth Rose  Paul, Kimberly C.  Lee, Eunice  Jerrett, Michael  Su, Jason  Wu, Jun  Shih, I. Fan  Haan, Mary  Ritz, Beate | 2020 | USA | 1,612 Mexican–American participants from the Sacramento Area Latino Study on Aging were followed every 12–15 months via home visits from 1998 to 2007. | Risk of dementia | N/A | Neighborhoods | To investigate the association between local traffic-related noise pollution and incident dementia or CIND^18^ during a 10-year follow-up period. | Traffic-related noise exposure was associated with increased risk of dementia or CIND in elderly Mexican–Americans. |  | * |  |  |  | Some spatial analysis + noise estimation | Data preparation |
| 64 | The Area Deprivation Index: A novel tool for harmonizable risk assessment in Alzheimer’s disease research | Zuelsdorff, Megan  Larson, Jamie L.  Hunt, Jack F.V.  Kim, Alice J.  Koscik, Rebecca L.  Buckingham, William R.  Gleason, Carey E.  Johnson, Sterling C.  Asthana, Sanjay  Rissman, Robert A.  Bendlin, Barbara B.  Kind, Amy J.H. | 2020 | USA | Participants (N = 2119) were drawn from the Wisconsin Registry for Alzheimer's Prevention study (n = 1501) and the Wisconsin Alzheimer's Disease Research Center clinical core (n = 618). | The association between neighborhood disadvantage and cognitive function | N/A | Census track | To assess associations between block group–level neighborhood disadvantage and cognitive function across several specific domains in a community-based cognitive aging cohort. | 1) neighborhood disadvantage is associated with poorer cognitive function across multiple domains in middle-aged and older adults. 2) The findings also indicate that geocoded neighborhood disadvantage holds potential as a practical marker for research initiatives aiming to benefit communities at increased risk for cognitive dysfunction and ADRD^19^ |  | * |  |  |  | linkage and aggregation | Data preparation |
| 65 | Geographical Variation in Antipsychotic Drug Use in Elderly Patients with Dementia: A Nationwide Study | Zakarias, Johanne Købstrup  Jensen-Dahm, Christina  Nørgaard, Ane  Stevnsborg, Lea  Gasse, Christiane  Andersen, Bodil Gramkow  Søren, Jakobsen  Waldorff, Frans Boch  Moos, Torben  Waldemar, Gunhild | 2016 | Denmark | Antipsychotic drug use among elderly (≥65 years) with (n = 34,536) and without (n = 931,203) a dementia diagnosis across the five regions and 98 municipalities | Geographical variances antipsychotic drug use | N/A | municipality | To investigate potential geographical variances in use of antipsychotic drugs in dementia care. | a pronounced geographical variation in the use of antipsychotics among elderly patients with dementia that could not be explained by variations in age and sex. | * |  |  |  |  | Mapping | Disease mapping/  Surveillance,  Dementia care/Rehabilitation |
| 66 | Investigating spatial convergence of diagnosed dementia, depression and type 2 diabetes prevalence in West Adelaide, Australia | Aw, Jessica Yi Han  Smurthwaite, Kayla  Bagheri, Nasser | 2020 | Australia | Data from 16 general practices | Prevalence of diagnosed dementia, depression and type 2 diabetes patients | N/A | SA1 | To investigate the factors associated with, the spatial variation and spatial convergence of diagnosed cases of dementia, depression and T2D^20^ | multi-level analysis revealed shared associations between dementia, depression and T2D. Both depression and T2D were found to be significant in dementia diagnosis. Hyperlipidemia or hypertension diagnosis and belonging to lower socioeconomic status were significantly associated with diagnosed T2D and depression. The spatial distribution of each disease varied across west Adelaide. Spatial convergence of the three diseases was observed in some hot spot and cold spot clusters. | * | * |  |  | * | Mapping,  Getis-Ord Gi*,  linkage and aggregation | Disease mapping/  Surveillance (2),  Data preparation |
| 67 | Joint space–time Bayesian disease mapping via quantification of disease risk association | Baer, Daniel R  Lawson, Andrew B  Joseph, Jane E | 2021 | USA | All MCI and AD diagnoses for inpatient and ER visits for all payers at county levels | Alzheimer’s disease risk | N/A | County | To propose and evaluate novel BHMs^21^ for disease mapping which in order to improve in characterizing AD risk. Using novel models to spatiotemporal AD count data arising from the counties of SC^22^ as well as to simulated data | The improved Bayesian hierarchical models have improved goodness of fit in mapping dementia risk. | * |  |  |  | * | Mapping,  Spatio-temporal Bayesian models | Disease mapping/  Surveillance (2) |
| 68 | General Practice Clinical Data Help Identify Dementia Hotspots: A Novel Geospatial Analysis Approach | Bagheri, Nasser  Wangdi, Kinley  Cherbuin, Nicolas  Anstey, Kaarin J. | 2017 | Australia | Clinical records from 16 general practices (468 Statistical Area level 1 s, N = 14,746) from the city of west Adelaide, Australia | Dementia risk | N/A | SA1 | 1) To estimate the levels of dementia risk in the community using general practice data;  2) To assess spatial variation of dementia risk;  3) To identify risk clusters (hotspots) and their association with socioeconomic status. | 1)Significant hotspots were observed in eastern and southern areas and cold spots were observed in the western area within the study perimeter. 2)Significant hotspots were observed in low socio-economic communities. 3)dementia risk scores increased with age, sex (female), high cholesterol, no physical activity, living alone (widow, divorced, separated, or never married), and co-morbidities such as diabetes and depression Similarly, smoking was associated with a lower dementia risk score | * | * |  |  | * | Mapping,  Getis-Ord Gi*, Local Moran,  some spatial analysis | Disease mapping/  Surveillance (2),  Data preparation |
| 69 | A GPS-Based Framework for Understanding Outdoor Mobility Patterns of Older Adults with Dementia: An Exploratory Study | Bayat, Sayeh  Naglie, Gary  Rapoport, Mark J.  Stasiulis, Elaine  Widener, Michael J.  Mihailidis, Alex | 2021 | Canada | A total of 7 people with dementia (PwD) and 8 cognitively intact controls, aged 65 years or older | Outdoor mobility patterns | GPS | Individual | To develop a comprehensive framework for comparing outdoor mobility patterns of cognitively intact older adults and older adults with dementia using passively collected GPS data | The results identified several significant differences between the 2 groups. PwD participated in more medical-related and fewer sport-related activities compared to the cognitively intact CTLs^23^. longer duration of daily walking time and longer outdoor activities at night, after 8 p.m. are associated with cognitively intact individuals |  |  |  | * |  | GPS | Basic research |
| 70 | Health Disparities in the Relationship of Neighborhood Greenness to Mental Health Outcomes in 249,405 U.S. Medicare Beneficiaries | Brown, Scott C.  Perrino, Tatiana  Lombard, Joanna  Wang, Kefeng  Toro, Matthew  Rundek, Tatjana  Gutierrez, Carolina Marinovic  Dong, Chuanhui  Plater-Zyberk, Elizabeth  Nardi, Maria I.  Kardys, Jack  Szapocznik, José | 2018 | USA | A population-based sample of 249,405 U.S. Medicare beneficiaries aged ≥65 years living in Miami-Dade County, Florida, USA, whose location did not change from 2010 to 201 | Rate of mental health outcomes | N/A | Census block | To examine the association between block-level greenness (vegetative presence) and mental health outcomes (Alzheimer’s disease and depression) | Higher levels of greenness (as measured by mean NDVI^24^ at the Census block level) were associated with reduced odds of two mental health outcomes: Alzheimer’s disease and depression. Planned post-hoc analyses revealed that higher levels of greenness were associated with even greater mental health benefits in low-income neighborhoods |  | * |  |  |  | some spatial analysis | Data preparation |
| 71 | Association of Low-Level Ozone with Cognitive Decline in Older Adults | Cleary, Ekaterina Galkina  Cifuentes, Manuel  Grinstein, Georges  Brugge, Doug  Shea, Thomas B. | 2017 | USA | 5,116 participants aged 60 or more between 2005 and 2008 from an ongoing longitudinal study of ADC program participants compiled by the University of Washington’s National Alzheimer’s Coordinating Center | Cognitive decline rate | N/A | ZIP codes | To conduct a retrospective analysis of whether or not air pollution influences cognitive performance among participants of the national ADC program. | Increased levels of ozone correlated with an increased rate of cognitive decline, following adjustment for key individual and community level risk factors. individuals harboring one or more APOE4 alleles exhibited a faster rate of cognitive decline. In contrast to ozone, there was not any correlation between ambient PM2.5 and cognitive decline at regulatory limits | * | * |  |  | * | Mapping,  Spatio-temporal Bayesian models,  Over analysis, Interpolation | Disease mapping/  Surveillance (2),  Data preparation |
| 72 | Regional specific groundwater arsenic levels and neuropsychological functioning: a cross-sectional study | Edwards, Melissa  Johnson, Leigh  Mauer, Cortney  Barber, Robert  Hall, James  Obryant, Sid | 2015 | USA | 1390 participants (733 AD, 127 MCI, and 530 with normal cognition) enrolled in the Texas Alzheimer’s Research and Care Consortium. | The association between regional specific groundwater arsenic concentrations and neuropsychological functioning | N/A | ZIP codes | To examine the link between GIS-estimated regional specific groundwater levels and neuropsychological functioning in a sample of individuals with and without cognitive impairment. | Estimated regional groundwater arsenic concentrations were found to be negatively associated with neuropsychological performance. linking higher arsenic concentration levels with better performance on tasks related to global cognition MMSE^26^ | * | * |  |  |  | Mapping,  Interpolation | Disease mapping/  Surveillance, Data preparation |
| 73 | Alzheimer patient detection using Bayesian network model | Fei, Chong Tiam  Yusof, Umi Kalsom  Khalid, Mohd Nor Akmal | 2016 | Malaysia | Four data Alzheimer  Patients were  used for training, 13 data were used for testing of | Alzheimer  Patient Detection | GPS | Individual | To propose a tracking approach for Alzheimer patients by utilizing a Bayesian network classifier to classify sets of data and automate the tracking process | The proposed Bayesian network classifier in estimating the probability of wandering, is able to correctly output the expected results with high capability and accuracy. |  |  |  | * |  | GPS | Basic research |
| 74 | Development of Regional Disparities in Alzheimer’s Disease Mortality in the Slovak Republic from 1996 to 2015 | Gavurová, Beáta  Kováč, Viliam  Jarčušková, Dominika | 2018 | Slovakia | Data of the individual districts of the Slovak Republic to get a detailed spatial view and for each year of the explored period | Standardised Mortality rate of AD | N/A | District | To analysis the regional disparities of the standardized mortality rate of AD | The results reveal a very heterogeneous structure of the standardized mortality rate. | * |  |  |  |  | Mapping | Disease mapping/  Surveillance |
| 75 | Geographic Patterns of Parkinsonism-Dementia Complex on Guam | Zhang, Zhen Xin  Anderson, Dallas W.  Mantel, Nathan | 1990 | USA | The case registry on Guam was the source of incidence data. (19 election districts of Guam) | Parkinsonism-Dementia incidence | N/A | District | To investigate geographic and temporal patterns in the incidence of Parkinsonism-Dementia | An overview of the geographic distribution of average annual age-adjusted incidence rates for parkinsonism-dementia is created which shows the geographic variations. | * |  |  |  |  | Mapping | Disease mapping/  Surveillance |
| 76 | A 2.5-Year Longitudinal Assessment of Naturalistic Driving in Preclinical Alzheimer’s Disease | Roe, Catherine M.  Stout, Sarah H.  Rajasekar, Ganesh  Ances, Beau M.  Jones, Jessica M.  Head, Denise  Benzinger, Tammie L.S.Williams, Monique M.Davis, Jennifer Duncan  Ott, Brian R.Warren, David K.Babulal, Ganesh M. | 2019 | USA | Cognitively normal drivers (aged 65 + years) with (n = 10) and without preclinical AD (n = 10) for 2.5 years. | Naturalistic longitudinal driving behavior | GPS | Individual | To examine self-reported and naturalistic longitudinal driving behavior among persons with and without preclinical AD. | Changes in driving behavior appear in preclinical AD. Preclinical AD participants travel fewer days, to fewer places and they have smaller driving space with greater dependence on other drivers than participants without preclinical AD. |  |  |  | * |  | GPS | Basic research |
| 77 | Dementia in Newfoundland: identification of a geographical isolate? | Frecker, M. F. | 1991 | Canada | The data of the five dementia codings for the 191 deaths identified from 3656 in 1985 Newfoundland death certificates and for the 208 deaths from 3582 in 1986 death certificates. | Dementia prevalence | N/A | Census subdivision | 1) To identify from death certificates regions with an increased incidence of dementia mortality; and 2) to determine whether a previously observed excess of patients with Alzheimer disease originating from a small area could be confirmed in a survey of death certificates. | If the birthplace of individuals with deaths coded as dementia is noted, regional differences in dementia rates can be assessed. | * |  |  |  |  | Mapping | Disease mapping/  Surveillance |
| 78 | Disorientation detection by mining GPS trajectories for cognitively-impaired elders | Lin, Qiang  Zhang, Daqing  Connelly, Kay  Ni, Hongbo  Yu, Zhiwen  Zhou, Xingshe | 2015 | China | 10 individuals’ real-world GPS dataset | Disorientation detection | GPS | Individual | To propose a disorientation detection method to provide appropriate real-time assistive services to elders with dementia. | The developed method can achieve 95% detection rate of disorientation with less than 3% of false positives, based on properly chosen parameters and detect outliers in one’s GPS trajectories. |  |  |  | * |  | GPS | Dementia care/Rehabilitation |
| 79 | Location prediction using GPS trackers: Can machine learning help locate the missing people with dementia? | Wojtusiak, Janusz  Mogharab Nia, Reyhaneh | 2019 | USA | Sample of 337 devices with at least 14 days of recorded data | Location and movement patterns | GPS | Individual | To explore the possibility of using machine learning methods applied to data from GPS trackers to create individualized models that describe patterns of movement | The developed model achieved the best AUC (and accuracy) in predicting locations based on day, time, and extracted location and duration attributes. |  |  |  | * |  | GPS | Dementia care/Rehabilitation |
| 80 | Geographical associations between aluminum in drinking water and death rates with dementia (including Alzheimer's disease), Parkinson's disease and amyotrophic lateral sclerosis in Norway | Trond Peder Flaten | 1990 | Norway | Mortality data were provided by the Central Bureau of Statistics of Norway | Death rate with dementia | N/A | Municipality | To investigating the possible relationship between AD and aluminum in drinking water or environmental exposure to aluminum in general | Comparisons of maps indicate a geographical association between aluminum in drinking water and registered death rates with dementia | * |  |  |  |  | Mapping | Disease mapping/  Surveillance |
| 81 | A low-cost and autonomous tracking device for Alzheimer’s patients | Hegde, Niharika  Muralidhara, Shishir  Ashoka, D. V. | 2019 | India | Two AD patients test case | Real-time location tracking | Systems-based wearable device for real-time location tracking using GPS | Individual | To present a low-cost, autonomous, embedded systems-based wearable device for real-time location tracking using GPS and the concept of geo-fencing. | By this system an alert is sent whenever the patient moves out of a certain “safe zone” area and sends subsequent updates after every 5 min of such an event. The system supports caregivers of patients with early and moderate Alzheimer’s disease. |  |  |  | * |  | GPS | Dementia care/Rehabilitation |
| 82 | Effects of air pollution on dementia over Europe for present and future climate change scenarios | Guzmán, Patricia  Tarín-Carrasco, Patricia  Morales-Suárez-Varela, María  Jiménez-Guerrero, Pedro | 2021 | European continent | Europe population data | Incidence rate of AD and DU | N/A | Country | To quantify the incidence rate of dementia associated with air pollution over a target domain covering Europe | Both AD and DU^28^ have a larger sensitivity to the exposure to NO2 than to PM2.5, being NO2 associated with a higher incidence rate due to air pollution. | * | * |  |  |  | Mapping,  Interpolation | Disease mapping/  Surveillance,  Data preparation |
| 83 | Life space metrics of older adults with mild cognitive impairment and dementia recorded via geolocation data | Liddle, Jacki  Ireland, David  Krysinska, Karolina  Harrison, Fleur  Lamont, Robyn  Karunanithi, Mohan  Kang, Kristan  Reppermund, Simone  Sachdev, Perminder S.  Gustafsson, Louise  Brauer, Sandra  Pachana, Nancy A.  Brodaty, Henry | 2021 | Australia | Eighteen older adults mild cognitive impairment and dementia | Lifespace metrics | GPS and Bluetooth beacon | Individual | To explore the feasibility of technology-based lifespace measurement for older people with dementia and mild cognitive impairment, including the generation of lifespace metrics, and investigation of relationships with health and mobility status. | Significant relationships were found between lifespace metrics and concurrent driving status and anteceding scores on the sit-to-stand test |  |  |  | * |  | GPS | Basic research |
| 84 | Using a GPS Watch to Characterize Life-Space Mobility in Dementia: A Dyadic Case Study | Chung, Jane  Boyle, Joseph  Pretzer-Aboff, Ingrid  Knoefel, Janice  Young, Heather M.  Wheeler, David C. | 2021 | USA | A dyad included a 64-year-old man with early-onset Alzheimer’s disease, and his 62-year-old wife. | Life-space mobility | GPS | Individual | To explore the feasibility of using a GPS watch to measure life-space mobility of a Latino persons with dementia. | GPS data indicated that the PWD made outdoor trips regularly and was active socially, with day-to-day variations. This study demonstrated a use for wearable location tracking technology to support accurate LSM^29^ assessment in dementia that can inform nursing practice, policy, and research to promote well-being and delay functional deterioration in PWD. |  |  |  | * |  | GPS | Basic research |
| 85 | Evaluation of Naturalistic Driving Behavior Using In-Vehicle Monitoring Technology in Preclinical and Early Alzheimer’s Disease | Davis, Jennifer D.  Babulal, Ganesh M.  Papandonatos, George D.  Burke, Erin M.  Rosnick, Christopher B.  Ott, Brian R.  Roe, Catherine M. | 2020 | USA | Thirty-three drivers (aged 60+ years) | Naturalistic driving behavior | GPS datalogger | Individual | To describe unsafe driving behaviors in individuals with symptomatic early AD using G-force triggered video capture and compare the driving habits of these symptomatic AD drivers to two groups of cognitively normal drivers, those with and those without evidence of cerebral amyloidosis using a GPS datalogger | Results demonstrate the utility of electronic monitoring to identify potentially unsafe driving events in symptomatic and preclinical AD |  |  |  | * |  | GPS | Basic research |
| 86 | Neighborhood greenspace exposure as a protective factor in dementia risk among U.S. adults 75 years or older: a cohort study | Erik D. Slawsky, Anjum Hajat, Isaac C. Rhew, Helen Russette, Erin O. Semmens, Joel D. Kaufman, Cindy S. Leary, and Annette L. Fitzpatrick | 2022 | USA | 3047 participants aged 75 years and older | Risk of dementia | N/A | census tract | To examine the association of residential greenspace with risk of dementia among older adults | Compared to low residential greenspace, high residential greenspace was associated with a reduced risk of dementia |  | * |  |  |  | Buffer analysis | Data preparation |
| 87 | A national cohort study (2000–2018) of long-term air pollution exposure and incident dementia in older adults in the United States | Liuhua Shi, Kyle Steenland, Haomin Li, Pengfei Liu, Yuhan Zhang, Robert H. Lyles, Weeberb J. Requia, Sindana D. Ilango, Howard H. Chang, Thomas Wingo, Rodney J. Weber & Joel Schwartz | 2021 | USA | ~2.0 million incident dementia cases (N = 12,233,371; dementia cohort) and ~0.8 million incident AD cases (N = 12,456,447; AD cohort) | Dementia incident | N/A | Zip Code - Neighbourhood | To investigate the association of long-term exposure to ambient fine particulate matter (PM2.5), nitrogen dioxide (NO2), and ozone (O3) with dementia and AD incidence. | For both outcomes (AD and dementia incidence), concentration-response relationships for PM2.5 and NO2 were approximately linear. This study suggests that exposures to PM2.5 and NO2 are associated with incidence of dementia and AD. | * | * |  |  |  | Mapping-Interpolation, Extrapolation | Disease mapping/  Surveillance,  Data preparation |
| 88 | Long‑term exposure to residential greenness and neurodegenerative disease mortality among older adults: a 13‑year follow‑up cohort study | Lucía Rodriguez‑Loureiro1, Sylvie Gadeyne, Mariska Bauwelinck, Wouter Lefebvre, Charlotte Vanpoucke and Lidia Casas | 2022 | Belgium | 1,134,502 individuals aged 60 years or older | Neurodegenerative disease mortality | N/A | Census tract | To study the association between residential surrounding greenness and neurodegenerative disease mortality in older adults | Increment of surrounding greenness was associated with a 4–5% reduction in premature mortality from all neurodegenerative diseases, Alzheimer’s disease, vascular and unspecified dementia, potentially independent from air pollution. |  | * |  |  |  | Buffer analysis, kriging interpolation | Data preparation |
| 89 | Using GPS Tracking to Investigate Outdoor Navigation Patterns in Patients With Alzheimer Disease: Cross-sectional Study | Vaisakh Puthusseryppady, Sol Morrissey, Min Hane Aung, Gillian Coughlan, Martyn Patel, BMBCh and Michael Hornberger | 2022 | United Kingdom | 15 AD and 18 age-matched healthy controls | The outdoor navigation patterns | GPS tracking | Individuals | To understand the outdoor navigation patterns of patients with AD in different conditions and investigate whether patients with AD experienced spatial disorientation when navigating through environments with a high outdoor landmark density and complex road network structure | Patients with AD restrict the spatial and temporal extent of their outdoor navigation in the community to successfully reduce their perceived risk of spatial disorientation, when they were alone |  |  |  | * |  | GPS (trajectory mining analytical techniques) | Basic research |
| 90 | Sex and Gender Differences in Environmental Influences on Dementia Incidence in Germany, 2014–2019: An Observational Cohort Study Based on Health Claims Data | Daniel Krefta, Gabriele Doblhammer | 2022 | Germany | 250,000 people aged 70 years or older | Dementia incidence | N/A | Postal code | To examine the influence of regional characteristics on the incidence of dementia and explores sex and gender differences using individual-level health information and regional characteristics | Environmental characteristics related to wealth and health resources of a region influence the risk of dementia among the elderly. The people living in regions with the highest tertile of income and remaining life expectancy at age 60 had lower dementia risks. | * | * |  |  |  | Mapping -some spatial analysis | Disease mapping/  Surveillance,  Data preparation |
| 91 | Machine learning detects altered spatial navigation features in outdoor behaviour of Alzheimer’s disease patients | Abhirup Ghosh, Vaisakh Puthusseryppady, Dennis Chan, Cecilia Mascolo, Michael Hornberger | 2022 | United Kingdom | 15 AD patients and 18 control | Spatial metrics | GPS tracking | Individuals | To use data-driven machine learning approaches to explore spatial metrics within real life navigational traces that discriminate AD patients from controls | Patients significantly differed from controls on entropy, segment similarity, and distance from home |  |  |  | * |  | GPS | Basic research |
| 92 | Everyday built environments of care: Examining the socio-spatial relationalities of suburban neighbourhoods for people living with dementia | Samantha Biglieri, Jennifer Dean | 2021 | Canada | 7 persons with dementia | The socio-spatial relationalities of suburban neighbourhoods for PWD | GPS | Individuals | To investigate the socio-spatial relationality between PWD and their neighbourhoods. | This study demonstrated the ways in which PWD were living through adversity in the built environment (“hopeful adaptation”), revealing innovative practices and structural barriers in suburban areas. | * |  |  | * |  | Mapping -GPS | Disease mapping/  Surveillance,  Dementia care/Rehabilitation |

**Abbreviations:**

1. OLS: Ordinary Least Squares
2. AD: Alzheimer’s Disease
3. VaD: Vascular Dementia
4. OP: organophosphorus
5. CHOs: Community Health Organizations
6. CHNs: Community Health Networks
7. SA1: statistical area level 1
8. PWD: People with dementia
9. SLSJ: Saguenay-Lac-Saint-Jean
10. ALS: amyotrophic lateral sclerosis
11. MS: multiple sclerosis
12. RFID: Radio Frequency Identification
13. MRC CFAS: Medical Research Council Cognitive Function and Ageing Study
14. MCI: Cognitive Impairment
15. HC: healthy controls
16. OOHB: out-of-home behavior
17. PSD: Presenile Dementia patients
18. CIND: Cognitive Impairment without Dementia
19. ADRD: Alzheimer’s disease and related dementias
20. T2D: Type 2 Diabetes
21. BHMs: Bayesian hierarchical models
22. SC: South Carolina
23. CTLs: Cognitively intact controls
24. NDVI: Normalized Difference Vegetation Index
25. ADC: Alzheimer’s Disease Center
26. MMSE: Mental State Examination
27. AUC: Area under curve
28. DU: Dementia from Unspecified cause
29. LSM: Life-space mobility
