# Supplementary material for: The role of geographic information system and global positioning system in dementia care and research: a scoping review
Source: Int J Health Geogr. 2022 Aug 4;21:8. doi: 10.1186/s12942-022-00308-1 (PMC9354285; doi:10.1186/s12942-022-00308-1)
Supplement: Supplementary file 4 — Additional file 4. Techniques. [file 12942_2022_308_MOESM4_ESM.docx]

| **GIS**  **Methodology**  **GIS**  **Application** | **Thematic mapping** | **Spatial modelling & analysis** | **GIS/GPS tools** | **Space time clustering** |
| --- | --- | --- | --- | --- |
| **Mapping/Surveillance** | Mapping (45) | Empirical Bayes (1) |  | Getis-Ord Gi* (2) |
|  |  | Bayesian disease mapping (1) |  | Local Moran (5) |
|  |  |  |  | Global Moran (2) |
|  |  | Besag-York-Mollie model (1) |  | spatial scan statistic (2) |
|  |  | Spatial buffer analysis & overlay analysis (1) |  | Spatio-temporal Bayesian modelling (3) |
| **Dementia care** | Mapping (1) | Distance based analysis & geosimulation modelling (5) | GPS (12) |  |
| **Contextual and risk factor analysis** |  | OLS (2) | Remote sensing and imaging (1) |  |
|  |  | Multilevel spatial random-effects Cox proportional hazards mode (1) |  |  |
|  |  | Spatial buffer analysis (1) |  |  |
| **Basic research** |  |  | GPS (18) |  |
| **Data preparation** |  | Aerial photograph analysis (1) | Remote sensing and imaging (2) |  |
|  |  | Spatial buffer analysis (6) |  |  |
|  |  | Distance based analysis (1) |  |  |
|  |  | Interpolation / Extrapolation (7) |  |  |
|  |  | Overlay analysis (1) |  |  |
|  |  | Linkage/aggregation (7) |  |  |
|  |  | Land use regression (2) |  |  |
|  |  | Noise estimation (1) |  |  |
|  |  | space syntax analysis (1) |  |  |
| **Planning** |  | Distance based analysis |  |  |

Each study may apply more than one technique
